# Protein‐Polymer Dynamic Hydrogels Utilizing Native Ligand Binding for Injectable Therapeutic Delivery and 3D Printing

**DOI:** 10.1002/adhm.71810

**Published:** 2026-10-09

**Authors:** S. Cem Millik, Yonghui Wang, Kefan Song, Jessica M. Snyder, LeAnn Le, Lauren G. Brown, Shanny Hsuan Kuo, Ashleigh B. Theberge, Suzie H. Pun, Alshakim Nelson

**Affiliations:** ^1^ Department of Chemistry University of Washington Seattle Washington USA; ^2^ Department of Bioengineering University of Washington Seattle Washington USA; ^3^ Department of Comparative Medicine University of Washington Seattle Washington USA; ^4^ Molecular Engineering and Sciences Institute University of Washington Seattle Washington USA; ^5^ Department of Urology University of Washington Seattle Washington USA

**Keywords:** 3D printing, drug delivery, hydrogels, injectable, nanocolloids, nanoparticles, shear thinning

## Abstract

Dynamic hydrogels comprising networks formed from reversible interactions have shown utility in minimally invasive delivery of encapsulated therapeutics, as well as in extrusion‐based 3D printing of tissue‐mimetic structures. Here, we report dynamic nanocolloidal hydrogels formed from the co‐assembly of spheroidal hyperbranched poly(glycerol) (HPG) and soluble albumin nanoparticles (SANs). Functionalization of the HPG with palmitate (a natural ligand of serum albumin) appears to enable self‐association of the HPG, as well as its interaction with SANs, to form dynamic networks. The properties of these networks can be tuned by modifying the size of the SAN in addition to other formulation parameters. To highlight the utility of these dynamic hydrogels, we demonstrate (i) in vitro and in vivo release of a model protein therapeutic from these injectable materials, (ii) preliminarily evaluate biocompatibility of these materials in a murine model, and (iii) demonstrate extrusion‐based 3D printing using these materials.

## Graphical abstract

Dynamic nanocolloidal hydrogels were synthesized via the co‐assembly of ligand‐modified spheroidal hyperbranched poly(glycerol) (HPG) and soluble albumin nanoparticles (SANs). Co‐assembly occurred through self‐association of the HPG, as well as its interaction with SANs via native ligand binding. These dynamic hydrogels were found to be rheologically tunable, injectable, 3D printable, and showed short‐term tolerability in vivo, as well as promise for delivery of a model protein therapeutic.

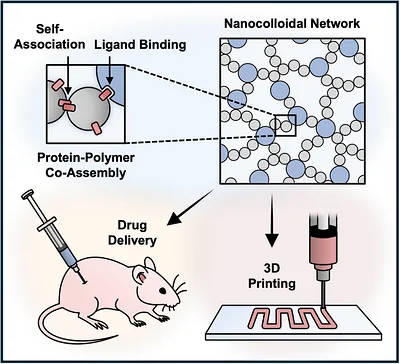

## 1 Introduction

Dynamic hydrogels have been widely investigated over the past few decades for applications such as injectable cell delivery, drug delivery, and 3D bioprinting [1, 2, 3, 4, 5, 6, 7, 8, 9]. Dynamic network structure gives these hydrogels complex viscoelasticity and makes them well‐suited for injection or extrusion [7, 8, 9, 10]. These materials can exhibit solid‐like behavior with dominant elastic character or liquid‐like behavior with dominant viscous character, depending on the magnitude of applied stress. Due to the reversible nature of the interactions that form these networks, transitions between these states are fully reversible via self‐healing. Under increased stress during extrusion, mechanical disruption of the dynamic network structure (i.e., yielding) causes a transition to a liquid‐like state, which allows flow from a syringe. During flow, viscosity decreases under shear (i.e., shear thinning and thixotropy) to facilitate injection/extrusion using minimal force. Following injection or deposition onto a surface, recovery of bulk solid‐like behavior facilitates retention of cells or therapeutic agents locally or affords fabricated constructs with stable form factors in the case of 3D printing [7, 8]. Thus, unlike their covalently cross‐linked or otherwise static counterparts, dynamic hydrogels respond to mechanical stimuli in ways that facilitate their patterning or delivery.

Dynamic hydrogels reported for injectable therapeutic delivery or extrusion‐based 3D printing (i.e., direct ink writing (DIW)) are typically composed of structural biopolymers and/or biocompatible synthetic polymers. Commonly used (bio)polymers include collagen, gelatin, fibrin, hyaluronic acid, alginate, cellulose derivatives, recombinant polypeptides, and poly(ethylene glycol)—with the majority of systems reported in the literature comprising multiple components [11, 12, 13, 14, 15, 16, 17, 18, 19, 20]. Dynamic hydrogels generally rely on ionic bonds, hydrophobic interactions, dynamic covalent bonds, specific biomolecular interactions, topological entanglements, or jamming for the formation of dynamic network structure [1, 2]. There is no widely agreed‐upon “gold‐standard” dynamic hydrogel for injection‐ or extrusion‐based applications in the biomedical space. This is perhaps reflective of the difficulty of designing a material that simultaneously possesses the complex rheological properties required for effective patterning or delivery, as well as the specific biological functionality required for the end use [5, 7, 8, 9, 21, 22, 23].

Dynamic hydrogel platforms that have shown promise for injection‐ or extrusion‐based applications in the biomedical space include the host–guest systems reported by Burdick and coworkers [4, 12, 14, 24, 25, 26, 27]. These gels feature dynamic networks formed from supramolecular self‐association of hyaluronic acid derivatized with cyclodextrin (host) and adamantane (guest) moieties. This platform has shown utility in 3D bioprinting and injectable therapeutic delivery [4, 25, 26, 27, 28]. As another notable example, the polymer–nanoparticle hydrogels reported by Appel and coworkers have shown promise for delivery of a wide scope of therapeutics, including small molecules, proteins, and cells [16, 29, 30, 31, 32]. These gels feature a dynamic network comprising polymeric core–shell nanoparticles physically cross‐linked via the association of hydrophobically derivatized cellulose derivatives with the nanoparticle cores.

Nanocolloidal hydrogels comprising networks formed from interactions between nanoparticle building blocks can exhibit favorable rheological properties for injection/extrusion [33, 34]. In some cases, nanocolloidal networks may consist solely of dispersed, interacting nanoparticles (including spheroidal polymers, proteins, or micelles); in other cases, additional small‐molecule or polymeric species may be incorporated to facilitate network formation [34]. Advantages of nanocolloidal hydrogels over traditional polymeric hydrogels can include favorable transport properties, distinct rheological properties, or functional attributes afforded by the high fraction of dispersed nanoparticles [34, 35, 36, 37]. Notably, nanoparticle building blocks exhibit minimal entanglement relative to linear polymers, which could be advantageous in dynamic systems that require high mobility of dissociated components to facilitate shear thinning, thixotropy, and self‐healing behaviors.

Protein‐based hydrogels can offer unique advantages derived from the structures and functions of their protein constituents. Furthermore, modern methods of protein engineering enable network design on the level of protein primary structure, which brings promise of unparalleled control over material functionality [38, 39, 40]. Advantages of protein‐based hydrogels can include specific biological functionality derived from biomolecular recognition, as well as useful properties related to material synthesis or processing [23, 41, 42]. Indeed, protein‐based hydrogels are also generally biocompatible and thus lend themselves well to use in a variety of bio‐interfacing applications [5, 23, 43, 44, 45]. For these reasons, greater understanding of methods for synthesizing hydrogel networks from functional protein building blocks is desirable.

Serum albumin is often considered a commodity protein; it is ubiquitous in research laboratories and as a component of many biomedical products. As the most abundant soluble protein found in vertebrates, serum albumin is easily purified in high yields and is thus available at relatively low cost [46]. In contrast with the notion that serum albumin is a simple commodity is the fact that it possesses sophisticated functionality. Perhaps most notably, serum albumin exhibits diverse binding affinity to a myriad of endogenous and exogenous substances [46, 47, 48, 49]. Among these substances are long‐chain fatty acids, to which albumin binds multivalently (≤ 6 M M^−1^) and with high affinity (*K*_A_ ∼ 1–69 × 10^7^ M^−1^) [46, 50]. Indeed, serum albumin is also biocompatible, enzymatically degradable, and highly aqueous‐soluble [46]. Overall, serum albumin is an abundant and accessible protein with numerous attributes that make it attractive for use as a biomaterial building block.

Here, we report dynamic nanocolloidal hydrogels comprising soluble albumin nanoparticles (SANs) and a spheroidal hyperbranched poly(glycerol) (HPG) derivatized minimally (< 1%) with palmitate moieties. Non‐covalent network formation in these hydrogels appears to rely on self‐association of the HPG via hydrophobic interactions between palmitate moieties as well as native palmitate binding of bovine serum albumin (BSA) units within the SANs. The proposed albumin–palmitate interaction, which could be competitively inhibited, proved particularly useful for tuning the rheological properties of these hydrogels. These gels exhibited reversible shear‐dependent solid‐to‐liquid (i.e., gel–sol) transitions, pronounced shear thinning, and prompt self‐healing upon removal of shear load. These gels could thus be conveniently mixed and dispensed from syringes with modest force, yet exhibited mechanical stability once injected or extruded. First, we report rheological evaluation and tuning of these materials toward effective application. Then, to highlight the utility of these materials, we demonstrate release of a model protein therapeutic from these gels in vitro and in vivo, we preliminarily evaluate their biocompatibility in vivo, and we demonstrate their 3D printability via DIW.

## 2 Results and Discussion

### 2.1 HPG as a Ligand‐Bearing Nanoparticle Building Block

We anticipated that given the constraint of utilizing a particular protein–ligand interaction, tunable and possibly high valency would be necessary for obtaining the specific rheological properties we desired. Thus, we selected HPG as a water‐soluble, nanoparticle building block with the capacity to display palmitate ligands with high valency. The hydrophilic surface functionality and spheroidal structure of HPGs (Figure 1a) additionally afford high aqueous solubilities and low intrinsic viscosities [51, 52, 53]. HPGs have also been shown to possess good biocompatibility in cell culture and in vivo [53, 54, 55, 56]. We adopted the approach of Kainthan et al. [52] to synthesize HPG with Z‐average hydrodynamic diameter *d*_H_ ≈ 14 nm and dispersity by DLS of ∼ 0.04 (Figure 1b).

**FIGURE 1:**
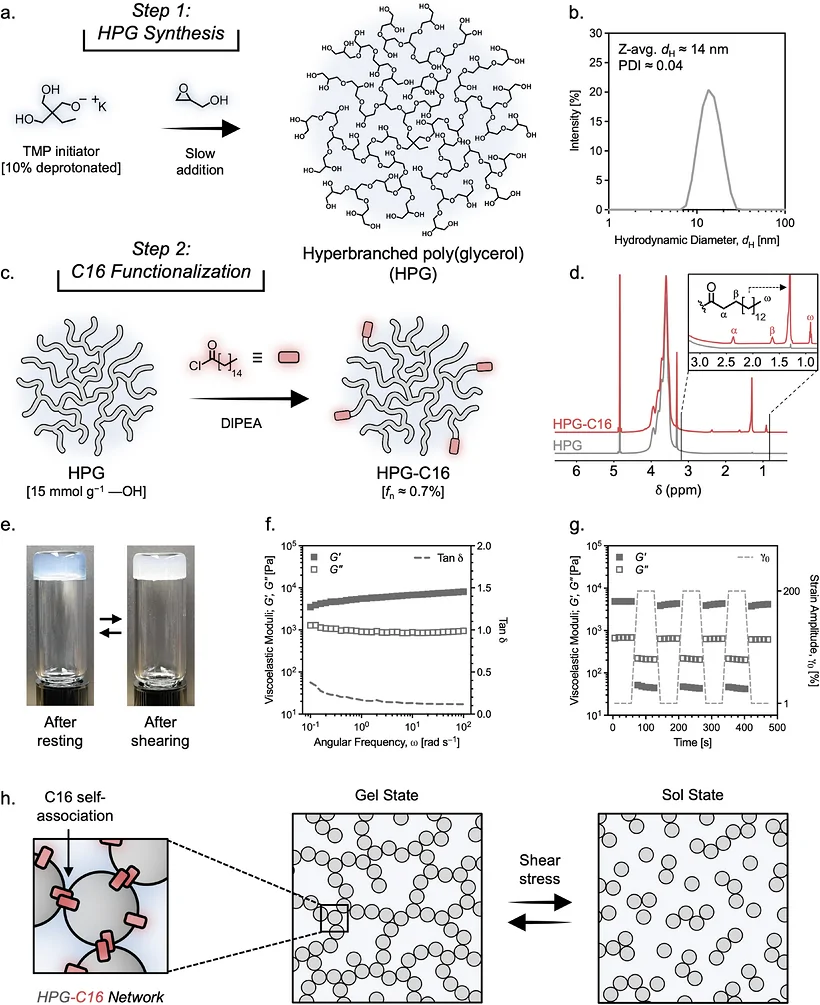
Synthesis and characterization of a palmitate‐modified hyperbranched poly(glycerol) (HPG) and its hydrogel. (a) Abbreviated synthetic scheme for HPG. (b) Dynamic light scattering (DLS) trace for HPG. A Z‐average hydrodynamic diameter *d*_H_ ≈ 14 nm was obtained, with a dispersity by DLS (PDI) of ∼ 0.04 (mean; *n* = 3). (c) Abbreviated synthetic scheme for HPG derivatized with palmitate moieties at ∼ 0.7% of hydroxyl groups (HPG‐C16). (d) ^1^H NMR spectra (500 MHz, CD_3_OD) of HPG and HPG‐C16. (e) Vial inversion test of 15 wt% HPG‐C16 hydrogel after several hours of rest versus minutes after shearing. (f) Frequency sweep data for 15 wt% HPG‐C16 hydrogel (mean; *n* = 3). (g) Multi‐step high‐ and low‐amplitude oscillatory tests showing self‐healing and thixotropy of 15 wt% HPG‐C16 hydrogel (mean; *n* = 3). (h) Conceptual depiction of proposed HPG‐C16 self‐association toward formation of a dynamic nanocolloidal network.

We hypothesized that HPG derivatized with palmitate moieties could exhibit physical association with serum albumin via the native long‐chain fatty acid binding activity of the protein. Furthermore, we anticipated that discrete interactions via the palmitate moieties, in the absence of substantial topological entanglements between the relatively compact HPG molecules, could afford materials that are more responsive to shear load. In aqueous mixtures, HPG derivatized with palmitate moieties at ∼ 0.7% of hydroxyl groups (HPG‐C16) appeared to self‐associate via hydrophobic interactions between the palmitate moieties (Figure 1c–h). Robust dynamic hydrogels could be obtained at ≥ 15 wt% polymer contents (Figure 1e–h). Small‐amplitude oscillatory shear experiments were performed to determine the strength and stiffness of a 15 wt% HPG‐C16 hydrogel, as well as the extent of its elastic character. This gel exhibited a storage modulus *G'* ≈ 6800 Pa, tan *δ* ≈ 0.13, and an apparent yield stress *σ*_y_ ≈ 130 Pa at angular frequency *ω* = 10 rad s^−1^ (Figure 1f,g and Figure S4). Notably, HPG‐C16 gels were opaque upon mixing but became nearly transparent after being allowed to rest. This change in opacity was reversible (Figure 1e).

In order to test whether co‐assembly of HPG‐C16 with serum albumin would occur, mixtures with a 1:1 mass ratio of HPG‐C16 to BSA were prepared. This ratio of components nominally affords ∼ 1.2 palmitate moieties per high‐affinity binding site of BSA [46]. Interestingly, mixtures of HPG‐C16 and BSA were nearly transparent, showing viscous‐liquid rather than gel character (Figure S5a,d,e). Increasing the amount of BSA in the mixture relative to that of HPG‐C16 failed to afford a gel, and in fact yielded mixtures that flowed more readily. Additionally, when unmodified HPG was analogously mixed with BSA for comparison, a decrease in viscosity of over 1000‐fold (at shear rate *γ* ^·^ = 1.0 s^−1^) was observed relative to the mixture with HPG‐C16 (Figure S6). In order to further probe the effects of the albumin‐palmitate interactions on the rheology of these materials, we performed a series of tests on mixtures of HPG‐C16 and BSA in which the BSA was pre‐saturated with free palmitate (Figure S5). The maximum palmitate loading achievable for BSA in vitro is considered to be 6 M M^−1^ [46]. Pre‐saturated BSA and HPG‐C16 mixtures were obtained simply by incubating a solution of BSA with an excess of powdered palmitate for 24 h [46], removing excess palmitate by filtration, and mixing the filtrate with HPG‐C16. These mixtures containing free palmitate competitively bound to BSA showed markedly different rheological behaviors than those without free palmitate. For pre‐saturated 1:1 mixtures at 15 wt% and 30 wt% total concentrations, frequency sweeps showed tan *δ* < 1 over the frequency range investigated, indicating gel‐like behavior (Figure S5d,e). Remarkably, for the pre‐saturated mixtures, curves for viscoelastic moduli *G'* and *G″* showed similar profiles to those of the pure HPG‐C16 gel (Figure 1f and Figure S5d,e). Steady‐shear curves also showed altered profiles when the BSA was pre‐saturated, bearing resemblance to those of the pure HPG‐C16 gel (Figures S4c and S5b,c). Notably, these mixtures containing free palmitate also appeared more opaque, with the 15 wt% mixture showing a similar appearance to that of the pure HPG‐C16 gel (Figure 1e and Figure S5a). Taken together, these findings supported the hypothesis that BSA was interacting with HPG‐C16 via the appended palmitate moieties. However, these interactions, which could be competitively inhibited, were not conducive to the formation of a physical network required for a self‐supporting hydrogel.

### 2.2 SANs Enable Productive Interactions toward Formation of Protein‐Polymer Networks

We posited that a nanoparticle with higher binding valency, in place of the native, predominantly monomeric BSA, could afford productive self‐assembly toward network formation. Palmer and coworkers have previously reported a simple and scalable approach for synthesizing covalently cross‐linked serum albumin species [57, 58, 59]. These species were obtained by cross‐linking albumin in dilute solution using glutaraldehyde. We adapted this approach to produce SANs in Z‐average hydrodynamic diameters *d*_H_ ≈ 30 nm (SAN‐30), *d*_H_ ≈ 45 nm (SAN‐45), and *d*_H_ ≈ 60 nm (SAN‐60), with dispersities by DLS ∼ 0.24–0.27 (Figure 2a–c). These SANs retained high aqueous solubilities and apparently low intrinsic viscosities reminiscent of native BSA. Circular dichroism (CD) spectroscopy of the SANs additionally showed retention of the characteristic α‐helical secondary structure of serum albumin (Figure 2d), suggesting that structurally intact BSA units within the SANs could retain capacity for ligand binding. Upon mixing SAN‐45 with HPG‐C16 in a 3:2 mass ratio, at 15 wt% total concentration, we observed gel formation (Figure 2e–h). Initially, excesses of the SANs over HPG‐C16 were included in formulations to account for the expected obstruction of a portion of fatty acid binding sites due to cross‐linking of the albumin. The 15 wt% 3:2 gel made with SAN‐45—abbreviated as 15‐(3:2)‐45—was transparent (Figure 2e) like the HPG‐C16 and BSA mixtures, possibly suggesting disruption of HPG‐C16 self‐association. Yet, the mixture containing the SAN showed tan *δ* < 1 over the frequency range investigated (Figure 2f), indicating dominant elastic character afforded by continuous network structure. This gel exhibited *G'* ≈ 2200 Pa, tan *δ* ≈ 0.42, and *σ*_y_ ≈ 270 Pa at *ω* = 10 rad s^−1^ (Figure 2f and Figure S8a,e,f). This gel also exhibited apparent viscosity *η* ≈ 630 Pa s at *γ* ^·^ = 1.0 s^−1^ and power‐law index *n* = 0.27 (Figure S8c,d). For comparison, when HPG‐C16 was replaced with unmodified HPG in a 15 wt% mixture with the SAN, a relatively low‐viscosity liquid resulted, exhibiting a decrease in viscosity of over 5000‐fold (at *γ* ^·^ = 1.0 s^−1^) relative to the mixture with HPG‐C16 (Figure S6). Finally, when SAN‐45 was pre‐saturated with free palmitate in a manner analogous to that for BSA, the palmitate‐saturated gel looked optically similar to the unsaturated gel (Figure S2); however, the saturated gel showed markedly different rheological behavior (Figure 3a–c and Figure S9). This result suggested that the SAN‐45 exhibited palmitate‐binding activity, and this interaction could be competitively inhibited—vide infra. We chose the 15‐(3:2)‐45 hydrogel as our starting formulation and proceeded to investigate the rheological effects of individually increasing or decreasing (i) the total concentration of solids, (ii) the mass ratio of the SAN to HPG‐C16, and (iii) the size of the SAN.

**FIGURE 2:**
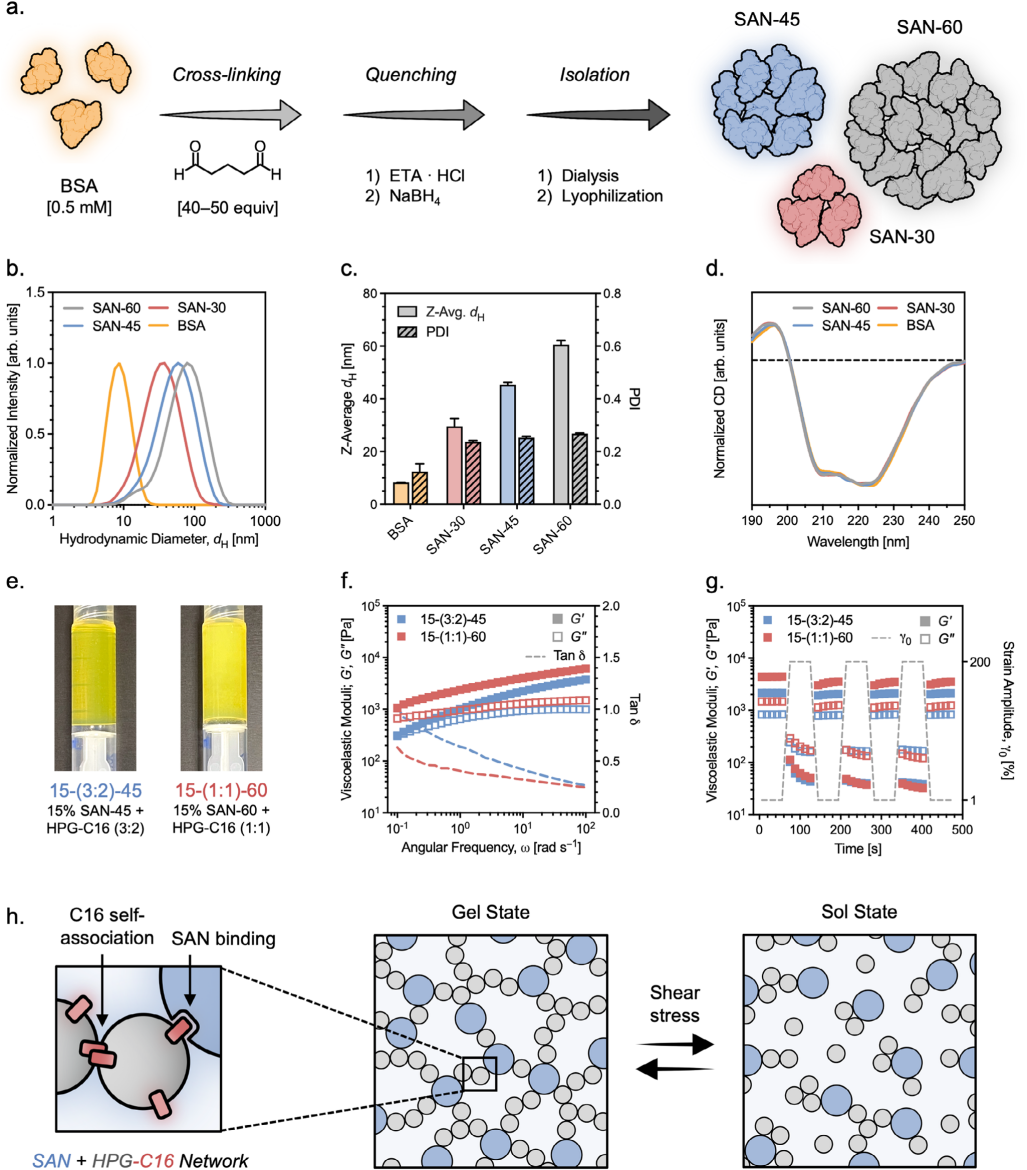
Synthesis and characterization of soluble albumin nanoparticles (SANs) and their protein‐polymer hydrogels. (a) Abbreviated synthetic scheme for SAN‐30, SAN‐45, and SAN‐60. (b) DLS traces for SANs and native BSA (mean; *n* = 3). (c) Comparison of *d*_H_ and dispersities by DLS (PDI) for SANs and native BSA (mean ± SD; *n* = 3). (d) Circular dichroism spectra for SANs and native BSA. (e) 15‐(3:2)‐45 and 15‐(1:1)‐60 hydrogels in syringes. (f) Frequency sweep data for 15‐(3:2)‐45 and 15‐(1:1)‐60 hydrogels (mean; *n* = 3). (g) Multi‐step high‐ and low‐amplitude oscillatory tests showing self‐healing and thixotropy of 15‐(3:2)‐45 and 15‐(1:1)‐60 hydrogels (mean; *n* = 3). (h) Conceptual depiction of proposed co‐assembly of HPG‐C16 and SAN toward formation of a dynamic nanocolloidal network.

**FIGURE 3:**
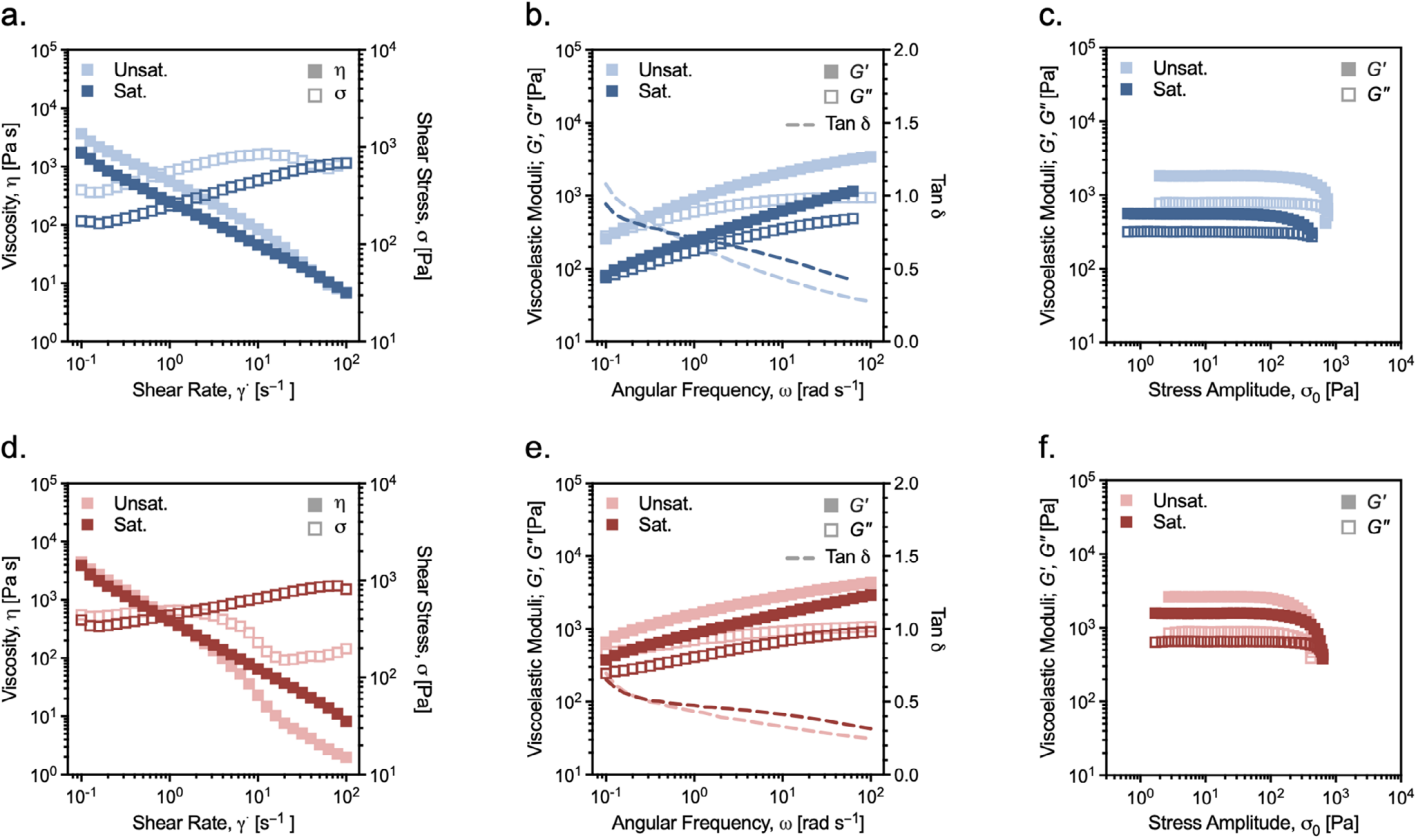
Rheometric data for protein‐polymer hydrogels with and without competitive inhibition. SANs were pre‐saturated with free palmitate (sat.) or left unsaturated (unsat.) prior to gel formulation. (a) Steady‐shear viscosity and stress curves for 15‐(3:2)‐45 gels (mean; *n* = 3). (b) Frequency sweep data for 15‐(3:2)‐45 gels (mean; *n* = 3). (c) Stress amplitude sweep data for 15‐(3:2)‐45 gels (mean; *n* = 3). (d) Steady‐shear viscosity and stress curves for 15‐(1:1)‐60 gels (mean; *n* = 3). (e) Frequency sweep data for 15‐(1:1)‐60 gels (mean; *n* = 3). (f) Stress amplitude sweep data for 15‐(1:1)‐60 gels (mean; *n* = 3).

### 2.3 Rheological Characterization and Tuning of Dynamic Nanocolloidal Hydrogels

For the investigation of the effects of varying total concentration, the mass ratio of the two components was kept constant at 3:2, and SAN‐45 was used in all formulations. We observed that a 10 wt% mixture appeared opaque and exhibited a weak‐gel character (Figure 4a–h and Figure S7e,h). Increasing total concentration to 20 wt% afforded a strong, transparent gel (Figure 4a–h; Figure S7e,h). The overall trends observed were that viscosity, storage modulus, and yield stress increased steadily with total concentration; however, *n* and tan *δ* did not change significantly between 10 wt% and 15 wt% and only showed statistically significant but slight reductions at 20 wt% (Figure 4b–h and Figure S7b,e,h). This result indicated that a slightly more elastic and more strongly shear‐thinning gel could be produced by increasing total concentration; however, these changes would be accompanied by a more prominent increase in the resistance of the material to being sheared or dispensed from a syringe. While we desired mechanical stability of the gel at rest, as well as strong shear‐thinning behavior, we found that the 20 wt% gel offered excessive resistance to mixing as well as injection/extrusion.

**FIGURE 4:**
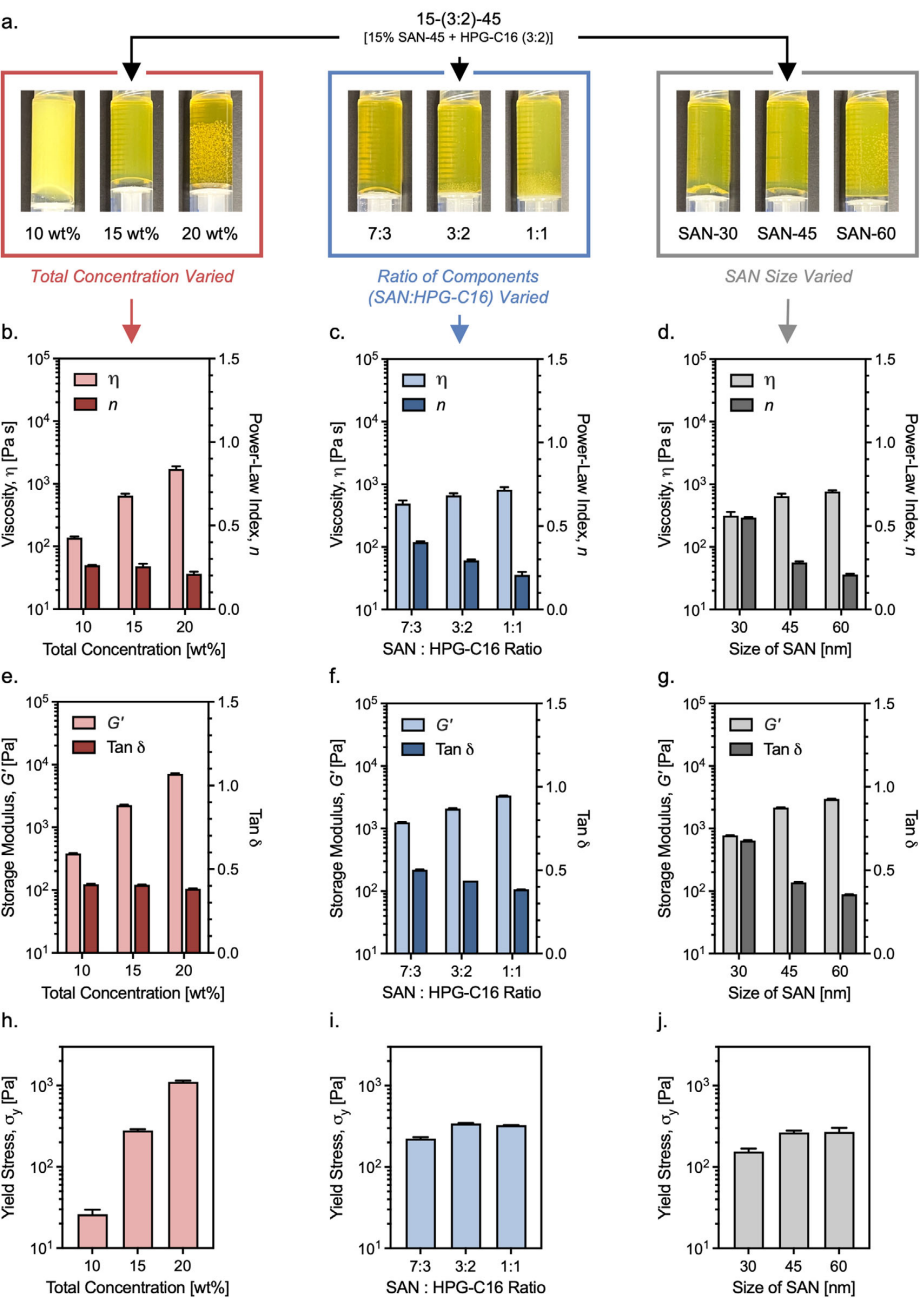
Summary data from systematic rheological evaluation of HPG‐C16 + SAN hydrogels with respect to total concentration, ratio of components, and SAN size. Bar charts show *η* at *γ^·^* = 1.0 s^−1^, *G'* and tan *δ* at *ω* = 10 rad s^−1^, and *σ*_y_ at *ω* = 10 rad s^−1^. (a) Tested hydrogels in syringes. The hydrogel in the center in each set of three gels is the 15‐(3:2)‐45 formulation. (b) Comparison of *η* and power‐law index *n* with total concentration varied (mean ± SD; *n* = 3). (c) Comparison of *η* and power‐law index *n* with ratio of components varied (mean ± SD; *n* = 3). (d) Comparison of *η* and power‐law index *n* with SAN size varied (mean ± SD; *n* = 3). (e) Comparison of *G'* and tan *δ* with total concentration varied (mean ± SD; *n* = 3). (f) Comparison of *G'* and tan *δ* with ratio of components varied (mean ± SD; *n* = 3). (g) Comparison of *G'* and tan *δ* with SAN size varied (mean ± SD; *n* = 3). (h) Comparison of *σ*_y_ for total concentration varied (mean ± SD; *n* = 3). (i) Comparison of *σ*_y_ for ratio of components varied (mean ± SD; *n* = 3). (j) Comparison of *σ*_y_ for SAN size varied (mean ± SD; *n* = 3).

Next, we investigated the effects of varying the mass ratio of the SAN to HPG‐C16. Total concentration was kept constant at 15 wt%, and SAN‐45 was used in all formulations. The three formulations tested, 7:3, 3:2, and 1:1 (SAN to HPG‐C16), were largely transparent (Figure 4a). Increasing elastic character, quantified by tan *δ*, and increasing extent of shear thinning, quantified by *n*, were apparent with increasing HPG‐C16 content (Figure 4c,f and Figure S7c,f). Changes in viscosity, storage modulus, and yield stress were not substantial, especially when increasing HPG‐C16 content from 40% of the total macromolecule content (in the 3:2 formulation) to 50% (in the 1:1 formulation) (Figure 4c–i and Figure S7c,f,i). Ultimately, the 7:3 and 3:2 formulations showed dominant viscous character (tan *δ* > 1) at low angular frequencies (Figure S7f) and flowed excessively at rest.

Finally, we investigated the effects of varying the SAN size. Total concentration was kept constant at 15 wt%, and the mass ratio of the two components was kept constant at 3:2. Again, the three formulations, made with SAN‐30, SAN‐45, or SAN‐60, were largely transparent (Figure 4a). The formulation with SAN‐30 showed viscoelastic‐liquid (as opposed to gel) character (Figure S7g). Between the SAN‐45 and SAN‐60 formulations, increasing the size of the SAN did not result in substantial changes in viscosity, storage modulus, and yield stress; however, moderate decreases in tan *δ* and *n* indicated increased elastic character and shear‐thinning behavior (Figure 4d–j and Figure S7d,g,j).

To obtain a hydrogel that could be conveniently mixed and dispensed from a syringe using modest force, yet would exhibit mechanical stability once injected or extruded from a nozzle, we decided to proceed with 15 wt% total concentration and a 1:1 mass ratio of SAN‐60 to HPG‐C16. This formulation is abbreviated as 15‐(1:1)‐60 (Figure 2e). This gel exhibited *G'* ≈ 3400 Pa, tan *δ* ≈ 0.33, and *σ*_y_ ≈ 170 Pa at *ω* = 10 rad s^−1^ (Figure 2f and Figure S8a,e,f). This gel also showed *η* ≈ 510 Pa s at *γ* ^·^ = 1.0 s^−1^ and *n* ≈ 0.03 (Figure S8c,d). The 15‐(1:1)‐60 gel was noticeably more stable at rest than the 15‐(3:2)‐45 gel but still exhibited good flowability under modest shear load.

To investigate the thixotropy and self‐healing behaviors of the 15‐(3:2)‐45 and 15‐(1:1)‐60 gels, we performed multi‐step oscillatory tests with alternating small‐amplitude (*γ*_·_ = 1%) and large‐amplitude (*γ*_·_ = 200%) steps. Both 15‐(3:2)‐45 and 15‐(1:1)‐60 gels exhibited time‐dependent disruption of their physical network structures at high strain amplitude, with prompt recovery of moduli during the subsequent low‐amplitude interval (Figure 2g and Figure S8h). The 15‐(1:1)‐60 gel exhibited slightly more pronounced thixotropic behavior, as evidenced by the more pronounced gradients of the modulus curves in both the high‐strain and low‐strain intervals (Figure 2g and Figure S8h). Finally, to investigate thermo‐rheological behaviors of these gels, temperature ramps between 5°C and 50°C were performed for both 15‐(3:2)‐45 and 15‐(1:1)‐60 gels. Both gels exhibited increasing tan *δ* and decreasing *G'* with increasing temperature. Nevertheless, both gels retained dominant elastic character over the temperature range investigated and showed fair mechanical stability at 37°C (Figure S8b,g).

Next, to investigate in greater detail the effects of competitive inhibition of HPG‐C16 binding of the SANs, we prepared 15‐(3:2)‐45 and 15‐(1:1)‐60 gels containing SANs pre‐saturated with palmitate. In general, during steady‐shear rheometry, gels containing non‐saturated SANs and HPG‐C16 exhibited edge fracture with occasionally visible sample displacement from the shear gap at high shear rates, due to viscoelastic instability. This is a signature of high elastic character at high shear rates or over short timescales [60]. In the steady‐shear curves for the 15‐(3:2)‐45 and 15‐(1:1)‐60 gels, viscoelastic instability is evidenced by minima in steady‐shear stress at moderate to high shear rates (Figure 3a,d). Interestingly, when SANs were pre‐saturated with free palmitate, both 15‐(3:2)‐45 and 15‐(1:1)‐60 gels could be sheared noticeably more smoothly, and signs of viscoelastic instability were absent from steady‐shear curves (Figure 3a,d). In addition, while tan *δ* at moderate to high frequencies was greater for both the pre‐saturated 15‐(3:2)‐45 gel and the pre‐saturated 15‐(1:1)‐60 gel (compared to unsaturated counterparts), tan *δ* at low frequencies was lower (Figure 3b,e and Figure S9b,e). For the 15‐(3:2)‐45 gel, pre‐saturation resulted in decreased viscosity, storage modulus, and yield stress (Figure 3a–c and Figure S9a–c). However, for the 15‐(1:1)‐60 gel, changes in viscosity, storage modulus, and yield stress were not substantial (Figure 3d–f and Figure S9d–f). These findings suggest a switch in network structure and dynamics with competitive inhibition of HPG‐C16 binding of SANs, which afforded materials that were simultaneously less elastic over short timescales yet slightly more stable at rest.

### 2.4 Injectability and Extrudability of Dynamic Nanocolloidal Hydrogels

Injection force measurements are a direct means of assessing clinically relevant rheology of injectable materials under high shear loads (Figure 5a and Figure S10). Forces < 20 N are preferred for clinical application of injectable formulations [8, 61, 62]. Additionally, quantification of injection/extrusion force can be valuable for assessing the 3D printability of a material via DIW [7]. To investigate the injectability/extrudability of these hydrogels, we performed force measurements using 1 mL syringes equipped with 23‐gauge and 27‐gauge needles of 0.5 in length (Figure 5b). All tests were performed at an injection rate of 1.2 mL min^−1^. Using 23‐gauge needles, the 15 wt% HPG‐C16 gel, the 15‐(3:2)‐45 gel, and the 15‐(1:1)‐60 gel all showed injection forces < 10 N (Figure 5c). Using 27‐gauge needles, the 15 wt% HPG‐C16 gel and 15‐(3:2)‐45 gel showed injection force < 20 N. The 15‐(1:1)‐60 gel showed slightly higher injection force at 22 N (Figure 5d). For comparison, a 30 wt% gel made from the commercially available non‐ionic surfactant, Pluronic F‐127, was tested. We and others have previously reported injection/extrusion of F‐127 hydrogels [63, 64, 65, 66, 67, 68, 69]. A disadvantage of F‐127 is that relatively high concentrations are required to obtain favorable rheological properties for injection/extrusion, which may compromise biocompatibility and other properties [70, 71, 72, 73]. The F‐127 gel showed an injection force of ∼ 15 N through the 23‐gauge needle (Figure 5c); however, the injection force increased beyond clinically relevant levels to ∼ 66 N when the 27‐gauge needle was used (Figure 5d).

**FIGURE 5:**
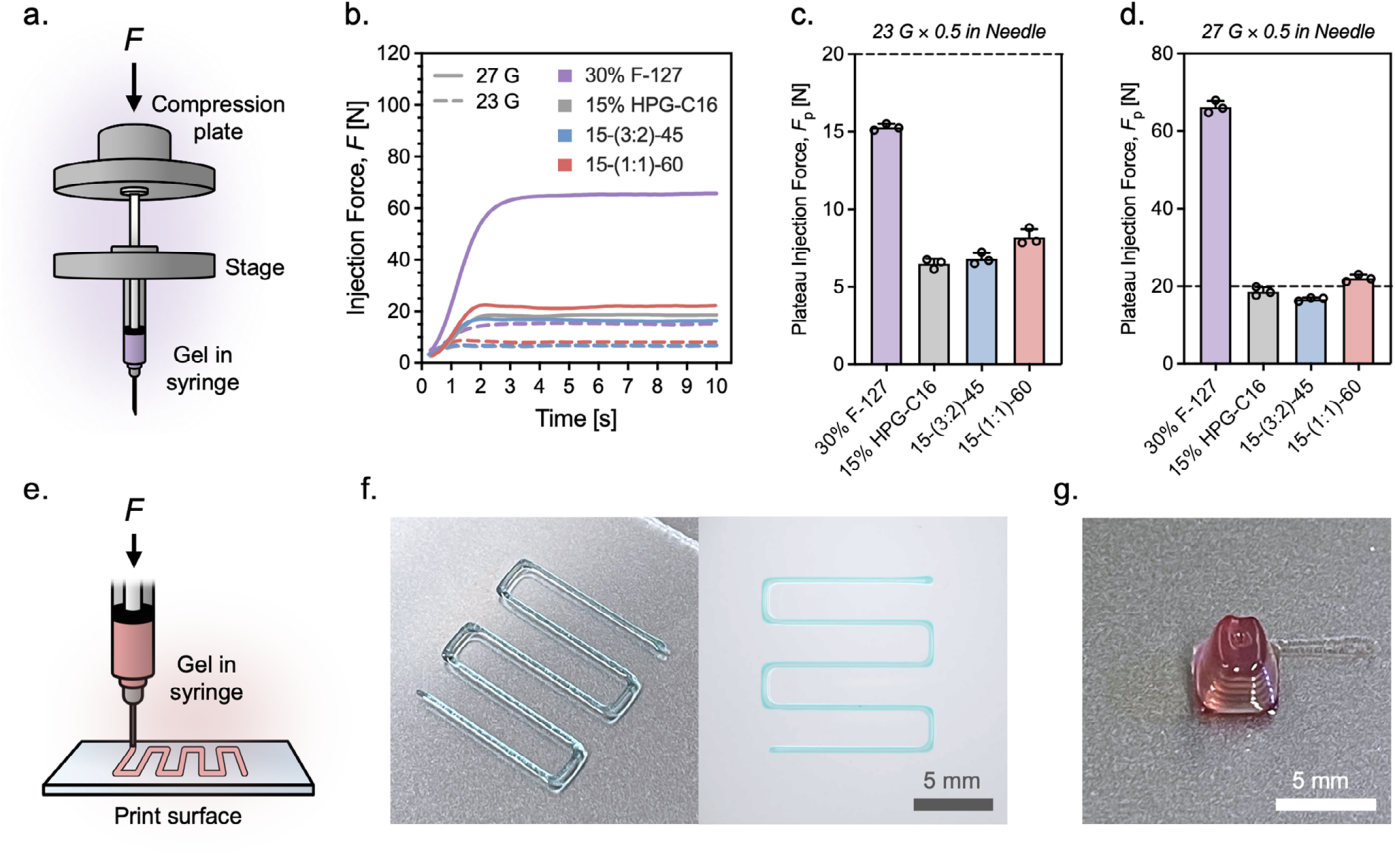
Injection force measurement and direct‐ink‐writing (DIW) 3D printing of hydrogels. Injection force data for 30 wt% commercially available F‐127 gel were included for comparison. (a) Schematic representation of injection force measurement using a load frame. (b) Injection force curves were obtained using 1 mL syringes with 0.5 in needles (23 and 27 G), using a 1.2 mL min^−1^ injection rate (mean; *n* = 3). (c) Comparison of plateau injection force *F*_p_ using a 23 G needle (mean ± SD; *n* = 3). (d) Comparison of *F*_p_ using a 27 G needle (mean ± SD; *n* = 3). (e) Schematic representation of DIW 3D printing. (f) Single‐layered print using 15‐(3:2)‐45 gel (dyed blue). (g) Multi‐layered print using 15‐(1:1)‐60 gel (dyed maroon).

To additionally demonstrate extrudability of these dynamic hydrogels toward 3D printing, we used DIW to pattern a serpentine structure using the 15‐(3:2)‐45 formulation (Figure 5e,f). This material exhibited smooth and consistent extrusion but was not ideal for patterning multiple layers without the use of a support material or secondary cross‐linking. The 15‐(1:1)‐60 formulation was better suited for printing multi‐layered constructs. Using the 15‐(1:1)‐60 formulation, we were able to print a cuboidal structure; however, some distortion of the structure after several minutes was observed (Figure 5g).

### 2.5 Gels Exhibit High Stability and Slow Release of Encapsulated Cargo

Interestingly, all investigated mixtures containing some portion of HPG‐C16 showed limited solubility in excess aqueous media. Despite the fact that hydrogels prepared with HPG‐C16 appeared homogeneous and nearly transparent, when these gels were incubated in excess aqueous solution, they appeared to exhibit further phase separation (Figure 6a). The same phenomenon was observed at both 21°C and 37°C. Thus, to explore dissolution of these materials in relevant aqueous media, we performed dissolution experiments over a period of 1 month (Figure 6b–d). Tests were performed under static conditions at 37°C. When 1X phosphate‐buffered saline (PBS) was used as the dissolution medium, the materials showed dissolution profiles characteristic of a first‐order process (Figure 6c). In this case, the 15 wt% HPG‐C16 gel exhibited the least mass loss, at 6.4% over 31.5 d. For comparison, a 15 wt% 1:1 mixture of monomeric BSA and HPG‐C16 exhibited the greatest mass loss, at 53% over 31.5 d. The 15‐(3:2)‐45 and 15‐(1:1)‐60 gels exhibited moderate to low degrees of mass loss, at 24% and 15%, respectively, over 31.5 d (Figure 6b,c). We hypothesized that serum components present in cell culture applications or in vivo could accelerate dissolution of these materials by potentially competing with binding interactions or otherwise solubilizing the network components. Therefore, we conducted an analogous experiment using Dulbecco's Modified Eagle's Medium (DMEM) supplemented with 10% fetal bovine serum (FBS) as the dissolution medium. Relative to results with 1X PBS, we observed similar degrees of mass loss in the serum‐containing DMEM until the 15.5 d time point. Interestingly, at the 31.5 d time point, mass loss was markedly increased (Figure 6b–d). The 15 wt% 1:1 mixture of monomeric BSA and HPG‐C16 exhibited virtually complete dissolution over this period. Surprisingly, the 15 wt% HPG‐C16 gel exhibited higher mass loss than the SAN‐containing gels, at 87% mass loss over 31.5 d. The 15‐(3:2)‐45 and 15‐(1:1)‐60 gels exhibited 71% and 40% mass loss, respectively, over 31.5 d (Figure 6b,d). These results suggested that SAN incorporation stabilized these gels against dissolution in serum‐containing culture medium.

**FIGURE 6:**
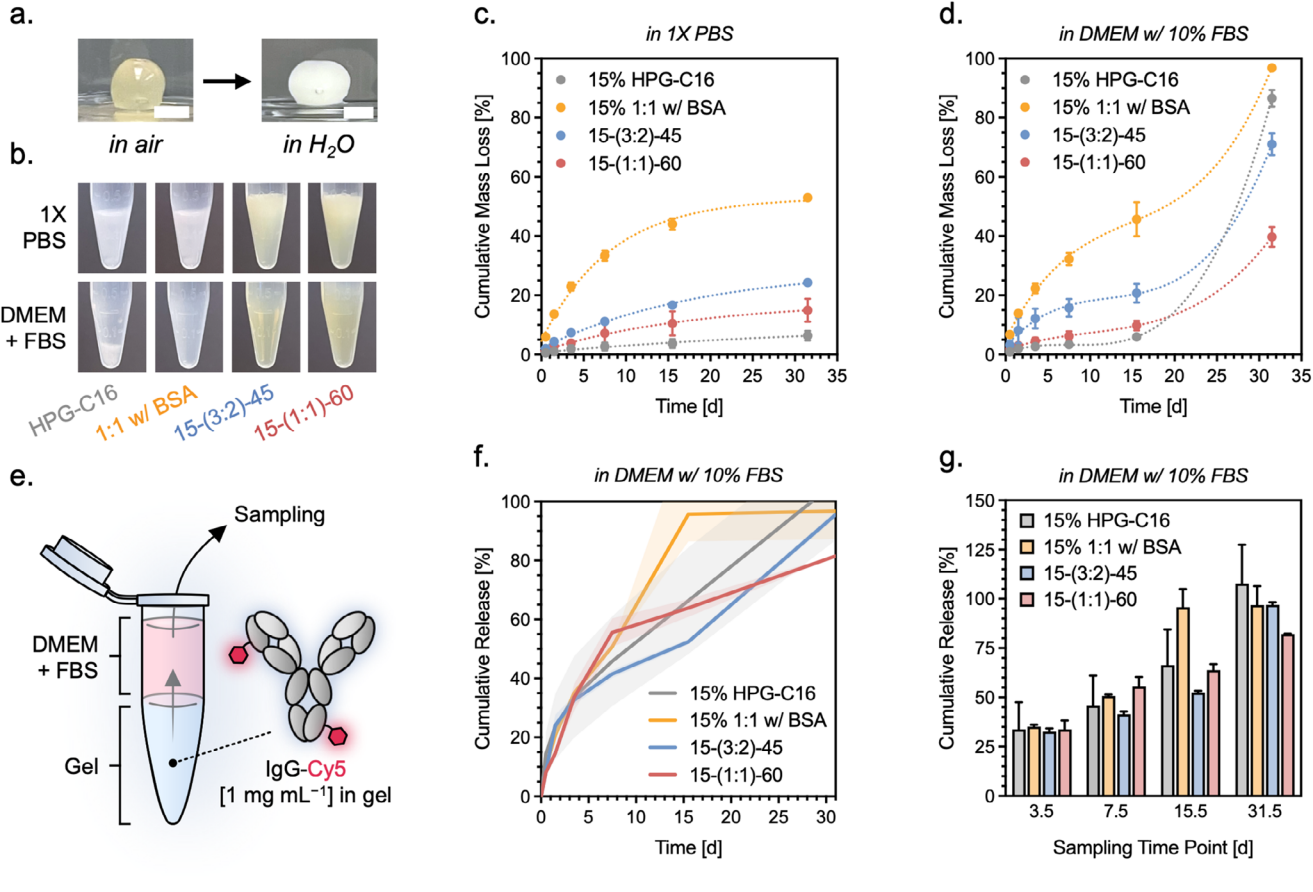
Aqueous stability of hydrogels and their in vitro release of a model protein therapeutic. Immunoglobulin G (IgG) was incorporated as the model protein therapeutic. (a) SAN + HPG‐C16 gel before and after immersion in water. Scale bars = 2 mm. (b) Different materials after 31.5 d in dissolution medium (1X PBS or DMEM with 10% FBS) at 37°C. (c) Cumulative mass loss of the materials over 31.5 d in 1X PBS at 37°C (mean ± SD; *n* = 3). (d) Cumulative mass loss of the materials over 31.5 d in DMEM with 10% FBS at 37°C (mean ± SD; *n* = 3). (e) Schematic representation of in vitro IgG release experiments. (f) Cumulative release of IgG from the materials over 31.5 d in DMEM with 10% FBS at 37°C (mean ± SD; *n* = 3). (g) Comparison of cumulative release of IgG from the materials at 3.5 d, 7.5 d, 15.5 d, and 31.5 d (mean ± SD; *n* = 3).

Next, to demonstrate in vitro release of a model protein therapeutic from these hydrogels, we prepared gels containing 1 mg mL^−1^ bovine immunoglobulin G (IgG) labeled with a sulfo‐Cyanine5 dye. Tests were performed under static conditions at 37°C. The release medium was DMEM with 10% FBS (Figure 6e). Release of IgG was tracked over 31.5 d by measuring fluorescence of the Cyanine5 dye relative to a standard curve. The 15 wt% HPG‐C16 gel, the 15‐(3:2)‐45 gel, and the 15 wt% 1:1 mixture of monomeric BSA and HPG‐C16 each exhibited virtually full release of the IgG cargo over the 31.5 d period. The 15 wt% 1:1 mixture of monomeric BSA and HPG‐C16 exhibited more rapid release than the other materials, at ∼ 95% after only 15.5 d. The 15‐(1:1)‐60 gel exhibited the least rapid release at ∼ 80% over 31.5 d (Figure 6f,g).

### 2.6 Release of a Model Therapeutic and Biocompatibility of Gels In Vivo

After obtaining promising results from the in vitro dissolution and release experiments, we proceeded to examine the release of the IgG model therapeutic in vivo. Each hydrogel, containing 100 µg (1 mg mL^−1^) of fluorescently labeled IgG, was injected into the subcutaneous space on the right dorsal caudal flank of an SKH1‐Elite hairless mouse (one injection per animal) (Figure 7a). Hydrogels tested included 15 wt% HPG‐C16 (Group 2), 15‐(3:2)‐45 (Group 3), and 15‐(1:1)‐60 (Group 4). 1X PBS containing 100 µg (1 mg mL^−1^) fluorescently labeled IgG was included as a control (Group 1). Release of IgG was tracked over 21 d using in vivo fluorescence imaging (IVIS) (Figure 7a–c). For the 1X PBS control, epifluorescence at the injection site decreased to ∼ 20% of the maximum value 48 h after injection, and to ∼ 10% after 7 d. In contrast, for 15 wt% HPG‐C16 and 15‐(3:2)‐45 gels, epifluorescence at the injection site decreased only to ∼ 85%–90% of the maximum value 48 h after injection, and to ∼ 30% after 7 d. The 15‐(1:1)‐60 exhibited the least rapid reduction in epifluorescence at the injection site, with ∼ 95% of the maximum value observed after 48 h and ∼ 70% observed after 7 d. After 21 d, minimal epifluorescence remained for all gels, suggesting elimination of the IgG cargo, and possibly the hydrogels, from the injection site (Figure 7b,c and Figure S11). Over the 21 days following injection, no weight loss or other clinical signs of illness were noted in mice from any of the treatment groups. Mild weight loss (∼ 5% of initial body mass) was observed for the 1X PBS group (Figure 7d), the cause of which is unknown.

**FIGURE 7:**
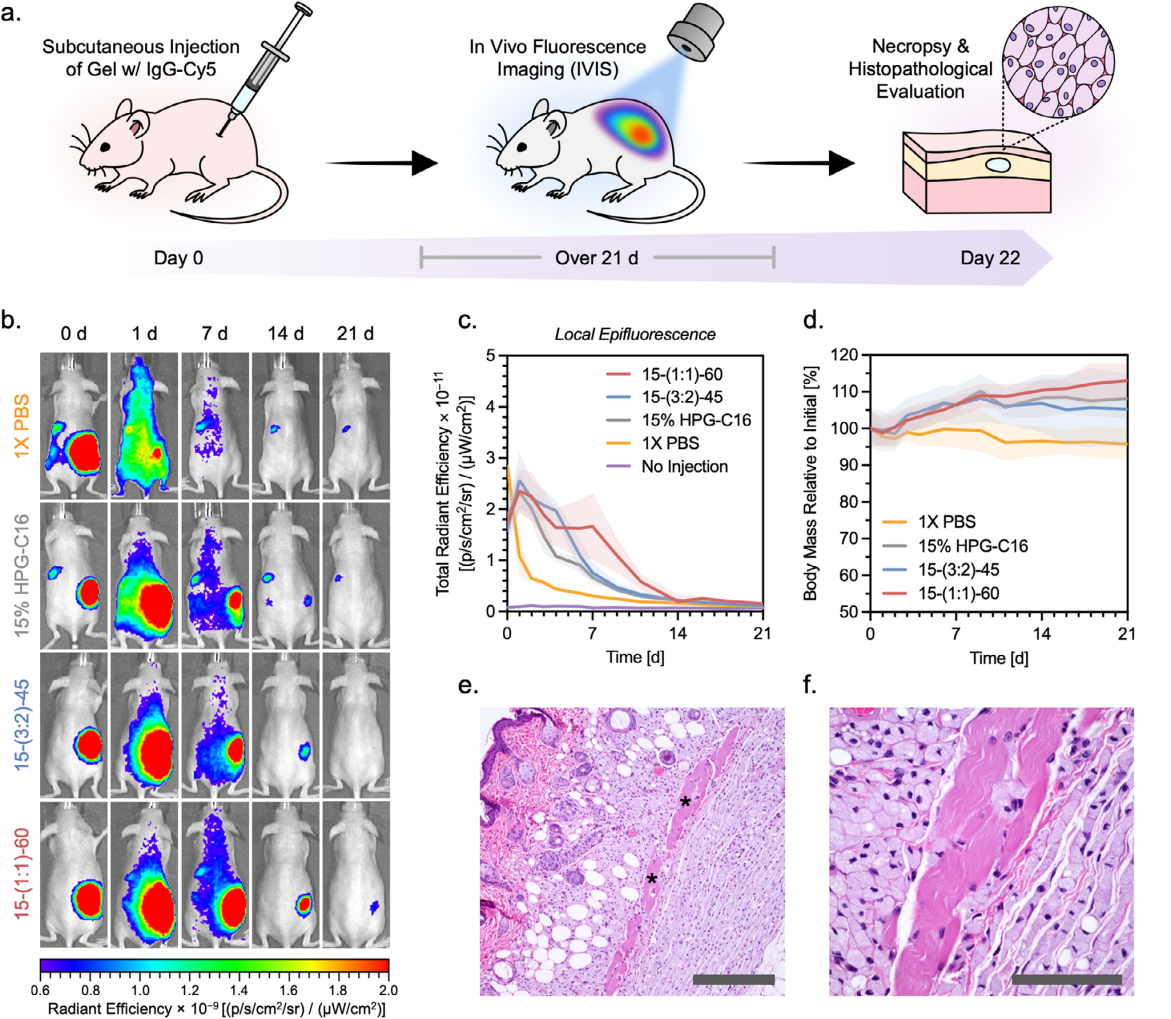
In vivo IgG release and preliminary evaluation of biocompatibility. (a) Schematic representation of in vivo IgG release experiments and biocompatibility testing of dynamic nanocolloidal hydrogels. (b) Representative in vivo fluorescence (IVIS) images showing IgG release over 21 d from subcutaneously injected gels in the right dorsal caudal flanks of mice. (c) Quantification of total radiant efficiency from the injection site over 21 d (mean ± SD; *n* = 4). (d) Body mass (relative to initial body mass) over 21 d for mice injected with different materials (mean ± SD; *n* = 4). (e) Representative low‐magnification (100×) image of skin showing infiltration of the tissue above and below the striated muscle (panniculus carnosus, asterisk) by histiocytes with abundant granular cytoplasm. Taken from Animal 2 of Group 2. Stained with hematoxylin and eosin (H&E). Scale bar = 250 µm. (f) Representative higher‐magnification (400×) view of histiocytes with granular cytoplasm. Taken from Animal 2 of Group 2. Stained with H&E. Scale bar = 100 µm.

In order to further assess potential local tissue response to the hydrogels, as well as potential systemic toxicity, animals were euthanized on day 22 by CO_2_ asphyxiation, blood was collected for complete blood count and serum chemistry, gross necropsies were performed, and tissues were collected for histopathological evaluation (Figure 7a). At necropsy, all mice had normal body condition scores of 3/5, except for two mice in Group 1, which had body condition scores of 2/5. Mice in Group 1 also had lower body mass than those in Groups 2–4 (Figure 7d and Table S1). No gross abnormalities or remnant hydrogel materials were appreciated at the injection site (i.e., subcutaneous tissue and skin over the right dorsal caudal flank) in any mouse. The only gross findings were mild enlargement of the spleen in hydrogel‐treated mice compared to control mice (2/4 mice in Group 2, 3/4 mice in Group 3, and 4/4 mice in Group 4) and mild mottling of the lung in mice from all groups, including Group 1, which is thought to be related to the method of euthanasia. No consistent histologic abnormalities were identified in the lung, heart, liver, kidney, spleen, or mesenteric fat of mice from any group to suggest systemic toxicity of the hydrogel. There was mild increased extramedullary hematopoiesis in the spleens of some mice from Groups 2, 3, and 4, which may have contributed to the increased size noticed grossly. One mouse from Group 3 had an inflammatory lesion at the heart base, which was considered more likely to be associated with an unrelated condition such as arteritis and unlikely to be associated with hydrogel treatment. The most pronounced histologic finding in all animals receiving a hydrogel injection was marked (Group 2) or moderate (Group 3 and Group 4) infiltration of macrophages with granular cytoplasm within the subcutaneous injection site and extending to involve the deep dermis to a lesser extent (Figure 7e,f and Table S2). There was no obvious associated neutrophilic inflammation, necrosis, or hemorrhage. Neutrophils and multinucleated giant cells were present in the dermis in all groups to a similar degree, often centered on hair fragments and follicles and most likely related to a background strain effect in hairless mice [74]. This influx of macrophages in the hydrogel‐treated mice is likely related to the response to and/or clearance of the injected hydrogels, as macrophages are important in phagocytosis and host response to biomaterials [75]. Results of complete blood counts and serum chemistries are presented in Table S3 and showed no consistent abnormalities other than a mild increase in alanine transaminase (ALT) and aspartate transaminase (AST) activities in Group 3, and a mild decrease in albumin and total protein in Group 4, relative to Group 1.

## 3 Conclusions

Dynamic hydrogels, which exhibit solid‐like behavior at rest but can be injected or 3D printed via stress‐induced flow from a syringe, are valuable in therapeutic delivery and bio‐fabrication applications. Relatively few examples of dynamic hydrogels that utilize biomolecular recognition in the network design currently exist in the literature. To our knowledge, all previous reports of dynamic hydrogels that incorporate specific protein or peptide interactions toward network formation feature engineered peptides or recombinant proteins, which may limit their scalability or accessibility. Here, we have exploited the long‐chain fatty acid binding functionality of one of the most readily available proteins, BSA, for the synthesis and rheological tuning of novel injectable/extrudable hydrogels. We have also highlighted the utility of HPG as a single‐molecule nanoparticle for presenting ligands to serum albumin species toward nanocolloidal network formation, as well as in the formation of pure HPG nanocolloidal networks. Finally, we have preliminarily evaluated the injectability of these hydrogels, their dissolution behavior, their ability to gradually release a model protein therapeutic in vitro and in vivo, their biocompatibility in vivo, and their promise for DIW 3D printing. Overall, while the hydrophobically derivatized HPG alone afforded robust dynamic hydrogels, incorporation of the albumin nanoparticles allowed further tuning of the rheological properties of the hydrogels, increased their stability in serum‐containing culture medium, and prolonged the release of encapsulated cargo in vivo. Future work will be aimed at further tuning these materials for specific applications in therapeutic delivery and bio‐fabrication.

## 4 Methods

### 4.1 Materials

Bovine serum albumin (BSA; BSAS 1.0) was purchased from Bovogen Biologicals. Immunoglobulin G from bovine serum (IgG; I5506), glycidol (G5809), glutaraldehyde solution (G7526; 8.7% w/w), calcium hydride (CaH_2_; 21170), 1,4‐dioxane (296309; anhydrous), *N*,*N*‐dimethylformamide (DMF; 227056; anhydrous), *N*‐methyl‐2‐pyrrolidone (NMP; 328634; anhydrous), 1,1,1‐tris(hydroxymethyl)propane (TMP; 148083; 99.8%), potassium methoxide solution (CH_3_OK; 60402; 25.1% in MeOH), palmitoyl chloride (P78; 98.6%), *N*,*N*‐diisopropylethylamine (DIPEA; D125806; 99.8%), sodium phosphate monobasic monohydrate (71507), sodium phosphate dibasic dihydrate (71643), ethanolamine (ETA; 398136), hydrochloric acid (HX0603), sodium borohydride (NaBH_4_; 213462), sodium azide (NaN_3_; S2002), Dowex 50WX8 hydrogen‐form resin (44509), acetone (179124), diethyl ether (Et_2_O; 673811), methanol‐*d*_4_ (CD_3_OD; 441384), Antifoam A concentrate (A5633), 3‐[(3‐cholamidopropyl)dimethylammonio]‐1‐propanesulfonate (CHAPS; 220201), and Pluronic F‐127 (F‐127; P2443) were purchased from MilliporeSigma. Molecular sieves (MK449004; grade 564) were purchased from VWR International. Methanol (MeOH; A452‐1), sodium chloride (S271), propylene glycol (ICN15195790), phosphate‐buffered saline 10X solution (PBS; BP399‐1), DMEM (31053028), and FluoroBrite DMEM (A1896701) were purchased from Fisher Scientific. Sulfo‐Cyanine5 NHS ester (NHS‐sulfo‐Cy5; 23320) was purchased from Lumiprobe.

### 4.2 Instrumentation

Proton nuclear magnetic resonance (^1^H NMR) spectra were obtained using a Bruker AVANCE series instrument operating at a 500 MHz frequency. Dynamic light scattering (DLS) data were obtained with a Zetasizer Nano ZS using a quartz cuvette (Starna Scientific). Rheometry was performed using a TA Instruments Discovery Hybrid Rheometer‐2 with an Advanced Peltier Plate system. Circular dichroism (CD) spectra were obtained with a Jasco J‐1500 CD spectrophotometer, using a quartz cuvette (FireflySci) with a 0.5 mm path length. Spectrophotometry (UV–vis) was performed using a Thermo Scientific NanoDrop One spectrophotometer and BioTek Cytation 5 cell imaging multimode reader. Fluorometry was performed using a BioTek Cytation 5. Injection force testing was performed using a TestResources 100 Series Universal Test Machine with 1.1 kN and 44 N load cells, compression fixtures, and a custom syringe holder (Figure S10). In vivo fluorescence imaging was performed using a Xenogen IVIS Spectrum.

### 4.3 Synthesis of Hyperbranched Poly(Glycerol) (HPG)

A more detailed description of HPG synthesis can be found in the Supporting Information. Briefly, TMP (0.450 g; 3.35 mmol) was added to a 250 mL 2‐neck Schlenk flask. MeOH (400 µL) was then added, followed by CH_3_OK solution (260 µL; 0.885 mmol; 25.1% in MeOH). After the TMP had dissolved, MeOH in the mixture was evaporated, and an overhead stirrer assembly was attached to the flask. Anhydrous dioxane (45 mL) was then charged to the flask. Using a syringe pump, glycidol (45 mL; 674 mmol) was added over 24 h at 115°C, under N_2_. Following glycidol addition, the reaction was allowed to proceed for an additional 24 h. Monomer consumption was confirmed by ^1^H NMR spectroscopy. Bulk dioxane was then aspirated, and MeOH (100 mL) was added to dissolve the product. Dowex 50WX8 hydrogen‐form resin (∼ 1 cc) was finally added to protonate the product. After 15 min, the Dowex resin was removed by centrifugation, and the product was precipitated in acetone. Precipitation in acetone was repeated once. The product was then dialyzed against deionized H_2_O. Following dialysis, the HPG solution was filtered with a 0.45 µm filter and stored at 5°C until use. Purity was confirmed by ^1^H NMR spectroscopy, and appropriate size was confirmed by DLS. Percent yield was ∼ 80%. ^1^H NMR (500 MHz, CD_3_OD, 25°C): *δ* = 3.94–3.53 (br; 1H, 2H; HPG).

### 4.4 Synthesis of Palmitate‐Modified HPG (HPG‐C16)

A more detailed description of HPG‐C16 synthesis can be found in the Supporting Information. Briefly, an aqueous solution of HPG (∼ 60 mL) was transferred to a 250 mL Schlenk flask and lyophilized. Trace water was removed under vacuum at 95°C. The mass of dry HPG was determined to be 14.8 g (222 mmol hydroxyl). Anhydrous NMP (100 mL) was charged to the flask to dissolve the HPG. DIPEA (510 µL; 2.93 mmol) was then added. Using a syringe pump, palmitoyl chloride solution (20.6 mL; 2.27 mmol; 3.33% v/v in anhydrous NMP) was added over 1 h at 21°C, under N_2_. The reaction was allowed to proceed for ∼ 20 h at 40°C before dialysis against MeOH. Following dialysis, the product was precipitated in acetone. Three additional precipitations were performed: once more in acetone and twice in Et_2_O. The product was finally dried under vacuum. Purity and functionalization (*f*_n_ = 0.70) were confirmed by ^1^H NMR spectroscopy. Appropriate size was confirmed by DLS. Percent yield was ∼ 95%. ^1^H NMR (500 MHz, CD_3_OD, 25°C): *δ* = 3.94–3.53 (br; 1H, 2H; HPG), 2.37 (br, 2H), 1.63 (br, 2H), 1.30 (br, 2H), 0.91 (t, 3H).

### 4.5 Synthesis of Soluble Albumin Nanoparticles (SANs)

A more detailed description of SAN synthesis can be found in the Supporting Information. The following procedure is a representative example, used to prepare SAN‐45. Briefly, BSA (2.00 g) was dissolved in 0.35 M phosphate buffer (58.69 mL; pH ∼ 7.4 at 37°C; *I* = 1.00 M, adjusted with NaCl) to afford a 0.5 mM BSA solution. The solution was filtered with a 0.22 µm syringe filter and added to a 250 mL round‐bottom flask. Antifoam A solution (20 µL; 25% w/w in propylene glycol) was then added, and the solution was stirred for 20 min at 37°C. Using a syringe pump, glutaraldehyde solution (8.7% w/w in H_2_O) was added over 3 h at 37°C. An addition rate of 0.425 mL h^−1^ (1.27 mL solution; 1.13 mmol glutaraldehyde) was used. Next, aqueous ethanolamine hydrochloride (4.50 mL; 11.3 mmol; 2.5 M) was added. The reaction was allowed to proceed for 1 h at 37°C. Aqueous NaBH_4_ (2.8 mL; 2.8 mmol; 1.0 M) was then added. After 30 min, an equivalent portion of NaBH_4_ was added, and the reaction was allowed to proceed for another 30 min. Finally, the reaction mixture was incubated at 5°C for ∼ 20 h. For purification, the reaction mixture was dialyzed against deionized H_2_O, filtered using a 1 µm syringe filter, and lyophilized. SANs were stored at −20°C or −80°C until use. Appropriate size was confirmed by DLS. Typical percent yield was ∼ 90%.

### 4.6 Dynamic Light Scattering (DLS)

For DLS of HPG, deionized H_2_O or MeOH was used as the dispersant. For DLS of HPG‐C16, MeOH was used as the dispersant. For DLS of BSA and SANs, 1X PBS was used as the dispersant. Samples were filtered with 0.22 µm cellulose acetate syringe filters. DLS experiments were run at 25°C, using a quartz cuvette. Experiments were conducted using a 173° non‐invasive backscatter configuration. Three measurements, consisting of 10 (10 s) runs each, were performed per sample and averaged. Normalized intensity was obtained by dividing each value in the dataset by the maximum value in the dataset.

### 4.7 Circular Dichroism (CD) Spectroscopy

SANs and BSA were dissolved in 25 mM phosphate buffer (pH ∼ 7.4) at a concentration of 0.25 mg mL^−1^. Samples were filtered with a 0.22 µm syringe filter (cellulose acetate). CD experiments were run at 25°C, using a quartz cuvette with a 0.5 mm path length. Spectra were obtained using a digital integration time of 4 s, a scanning speed of 20 nm min^−1^, an accumulation of 4 scans, and a bandwidth of 1 nm. The sample chamber was purged with N_2_ gas for 15 min after loading each sample, prior to running each experiment. A background spectrum for 25 mM phosphate buffer was recorded and subtracted from the spectrum for each material. Normalized spectra were obtained by dividing each value in the dataset by the square root of the sum of the squares of each value in the dataset.

### 4.8 Preparation of Hydrogels

A more detailed description of hydrogel preparation can be found in the Supporting Information. The following procedure is a representative example, used to prepare 2.0 g of a 15 wt% hydrogel with a 3:2 mass ratio of SAN‐45 to HPG‐C16—abbreviated 15‐(3:2)‐45. Briefly, HPG‐C16 (0.12 g) and SAN‐45 (0.18 g) were added as solids to one syringe. 1X PBS (1.7 mL) was added to a second syringe. The two syringes were then connected using an elbow connector, and their contents were dissolved and homogenized over 3 d at 5°C by occasionally pushing the contents back and forth between the two syringes. Excess air and small bubbles were removed by centrifugation. Hydrogels were stored at 5°C until use.

Pre‐saturation of SANs with palmitate was accomplished by preparing stock solutions of the SANs, mixing them with an excess of powdered palmitate (50 mg palmitate for ∼ 2.8 mL SAN solution), incubating the mixtures for 24 h, and filtering with a 0.22 µm syringe filter. Pre‐saturated SAN stock solutions were used to prepare the pre‐saturated SAN hydrogels. Standard (unsaturated) SAN hydrogels were prepared analogously but without free palmitate for direct comparison.

### 4.9 Rheometry of Hydrogels

A more detailed description of rheometry of hydrogels can be found in the Supporting Information. Hydrogels for rheometry were prepared as described above. A cross‐hatched 20 mm stainless steel geometry was used, with a cross‐hatched lower plate, and a solvent trap to minimize evaporation. Logarithmic shear rate sweeps in the range of 0.1–100 s^−1^ were performed at 25°C. Power‐law index *n* was determined by fitting a power‐law model to the manually selected power‐law region of each flow curve. Oscillatory strain amplitude sweeps in the range of 0.1%– 100% were performed at a constant angular frequency of 10 rad s^−1^, at 25°C. The limit of the linear viscoelastic regime in terms of stress amplitude was taken as the yield stress. Oscillatory frequency sweeps in the range of 0.1–100 rad s^−1^ were performed at a constant strain amplitude within the linear regime (1%), at 25°C. Multi‐step oscillatory tests, with alternating small‐amplitude (1%) and large‐amplitude (200%) steps (60 s), were performed at a constant angular frequency of 10 rad s^−1^, at 25°C. Tests with longer (600 s) steps were also performed. Finally, temperature ramps from 5°C to 50°C, at a rate of 2°C min^−1^, were performed at a constant strain amplitude of 1% and a constant angular frequency of 10 rad s^−1^. For all experiments, at least three runs were performed per sample and averaged.

### 4.10 Hydrogel Injection Force Measurements

A custom syringe holder (Figure S10) was designed and 3D printed to interface with the compression fixtures of a TestResources 100 Series Universal Test Machine, and hold a 1 mL syringe (BD, 309628) with the tip of the syringe oriented downward. Tests were performed with 23 and 27 G needles of 0.5 in length. Hydrogels were prepared as described above. A slight excess of hydrogel was loaded into the syringe prior to each test. A preload of 0.1 N was used for each test. An injection rate of 1.2 mL min^−1^ (68 mm min^−1^) was used over a test period of 10 s. For all experiments, three runs were performed and averaged. Plateau injection force *F*_p_ was determined by fitting an exponential plateau model to the injection force data (GraphPad Prism 10). Second‐order smoothing with 10 neighbors was applied to the injection force data for visualization (GraphPad Prism 10).

### 4.11 Direct Ink Writing (DIW)

Direct ink writing was performed on a HyRel Engine SR extrusion‐based 3D printer equipped with an SDS‐5 syringe dispenser. Standard 5 mL syringes (BD) were used with 27 G needles with 0.5 in length. An extrusion rate of 70 pulses µL^−1^, a print speed of 200 mm min^−1^, and a layer height of 0.21 mm were used for all prints.

### 4.12 Hydrogel Dissolution Experiments

Materials for dissolution experiments were prepared as described above. Each material was prepared using the corresponding dissolution medium. Dissolution media were 1X PBS and DMEM with 10% FBS and 1% penicillin–streptomycin. NaN_3_ (50 mM) was included in the dissolution media to prevent microbial contamination. Each material (300 mg) was loaded into a 1.5 mL microcentrifuge tube (VWR, 89000‐028) and centrifuged for 1 min at 3000 *g* to uniformly localize the material to the bottom of the tube. Dissolution medium (1.0 mL) was placed over each material. Tubes were incubated at 37°C without agitation. Time points for sampling were 0.5 d, 1.5 d, 3.5 d, 7.5 d, 15.5 d, and 31.5 d. At each time point, the supernatant was carefully collected from each tube and replaced with 1.0 mL of fresh dissolution medium. Collected supernatants were frozen with liquid N_2_ and lyophilized. Mass loss from materials due to dissolution was calculated from dry mass remaining after lyophilization, assuming concentrations of the components of the dissolution media remained constant. Three replicates were included for each material.

### 4.13 In Vitro IgG Release Experiments

A more detailed description of in vitro IgG release experiments can be found in the Supporting Information. Briefly, to prepare IgG‐Cy5, IgG solution (4.7 mL; 0.14 µmol) was diluted twofold with 0.1 M phosphate buffer (pH ∼ 7.4) with 20% v/v DMSO and 4% w/v CHAPS. NHS‐sulfo‐Cy5 solution (80 µL; 0.80 µmol; in DMF) was added and promptly mixed in. The reaction was allowed to proceed for 6 h at 21°C, with orbital shaking. The reaction mixture was finally refrigerated for 18 h, before the crude IgG‐Cy5 product was purified by diafiltration against 1X PBS with 10% v/v DMSO, followed by 1X PBS. The pure IgG‐Cy5 was concentrated to ∼ 5 mg mL^−1^ in 1X PBS and stored at 5°C until use within 3 days. Degree of labeling with the Cy5 dye was determined by spectrophotometry to be ∼ 2.0 M M^−1^. Percent yield was ∼ 90%.

Materials containing IgG‐Cy5 (1 mg mL^−1^) were prepared for in vitro release experiments, as described above, using 1X PBS with NaN_3_ (50 mM) to prevent microbial contamination. The release medium was FluoroBrite DMEM with 10% FBS, 1% penicillin‐streptomycin, and 50 mM NaN_3_. Each material (300 mg) was loaded into a 1.5 mL microcentrifuge tube (VWR, 89000‐028) and centrifuged for 1 min at 3000 *g* to uniformly localize the material to the bottom of the tube. Release medium (1.0 mL) was placed over each material. Tubes were incubated at 37°C without agitation. Time points for sampling were 0.5 day, 1.5 days, 3.5 days, 7.5 days, 15.5 days, and 31.5 days. At each time point, the supernatant was carefully collected from each tube and replaced with 1.0 mL of fresh release medium. Quantities of IgG‐Cy5 were determined by measuring fluorescence intensity of the supernatants and using a standard curve. Three replicates were included for each material.

### 4.14 In Vivo Release and Biocompatibility Experiments

A more detailed description of in vivo release and biocompatibility experiments can be found in the Supporting Information. For animal studies, hydrogels containing IgG‐Cy5 (1 mg mL^−1^) were prepared as described above, using sterile 1X PBS. Hydrogels and their components were prepared aseptically.

All animal studies were approved by, and performed in accordance with, the University of Washington Institutional Animal Care and Use Committee (protocol number: 4053‐01). Female SKH1‐Elite hairless mice (strain code: 477) aged 6–8 weeks were purchased from Charles River Laboratories. Four animals per group were subcutaneously injected, in the right dorsal caudal flanks, with 100 µL of a hydrogel or 1X PBS containing 100 µg of IgG‐Cy5. Mice were imaged (IVIS) and weighed once per day on days 0–4, 6, 7, 9, 11, 14, 16, 18, and 21. Epifluorescence from regions of interest was quantified in terms of radiant efficiency.

On day 22, mice were euthanized by CO_2_ asphyxiation, and cardiocentesis was performed for complete blood count and serum chemistry (three mice per treatment group). Following blood collection, gross necropsies were performed on four mice per group, and tissues were collected for histopathological evaluation by a board‐certified veterinary pathologist. Histology was performed on three mice per group (selected randomly). Tissues examined as part of the histopathological evaluation included skin and subcutaneous tissue harvested from the injection site, lung, heart, liver, kidney, spleen, and mesenteric fat with pancreas. Tissues were routinely paraffin‐embedded, sectioned at 4 µm, and slides were stained with hematoxylin and eosin.

### 4.15 Statistical Analysis

Data were reported as mean ± standard deviation (SD) unless specified otherwise. Normalized intensity for DLS was obtained by dividing each value in the dataset by the maximum value in the dataset. Normalized CD spectra were obtained by dividing each value in the dataset by the square root of the sum of the squares of each value in the dataset. Power–law index *n* was determined by fitting a power–law model to the manually selected power–law region of each flow curve. Ordinary one‐way ANOVA in conjunction with Tukey's multiple comparisons test with a single pooled variance was used to assess statistical significance in the summary rheometric data (GraphPad Prism 10). Statistical significance was defined as *p* < 0.05. Sample size (*n*) was three for each analysis. *F*_p_ was determined by fitting an exponential plateau model to the injection force data (GraphPad Prism 10). Second‐order smoothing with 10 neighbors was applied to the injection force data for visualization (GraphPad Prism 10).

## Author Contributions

**S. Cem Millik**: conceptualization, investigation, writing – original draft, methodology, formal analysis, data curation, and funding acquisition. **Yonghui Wang**: visualization, methodology, and investigation. **Kefan Song**: conceptualization, methodology. **Jessica M. Snyder**: investigation, visualization, formal analysis, and writing – review and editing. **LeAnn Le**: investigation, methodology. **Lauren G. Brown**: investigation, resources. **Shanny Hsuan Kuo**: investigation, visualization, formal analysis. **Ashleigh B. Theberge**: investigation, resources, and supervision, writing – review and editing. **Suzie H. Pun**: investigation, conceptualization, resources, supervision, funding acquisition, and writing – review and editing. **Alshakim Nelson**: conceptualization, funding acquisition, investigation, writing – review and editing, project administration, resources, and supervision.

## Funding

The authors acknowledge support from the University of Washington CoMotion Innovation Gap Fund (S2023‐8039‐Millik), the United States National Science Foundation Emerging Frontiers in Research and Innovation program (EFMA‐2223537), and the National Institutes of Health (NIH 1R01CA257563, NIH R35GM128648, and 5TL1TR002318‐08).

## Conflicts of Interest

A.B.T. reports receiving a gift to support research outside of the submitted work from Ionis Pharmaceuticals. A.B.T. has ownership in Seabright, LLC, which will advance new tools for diagnostics and clinical research with technology outside of the submitted work. The remaining authors declare no conflicts of interest.

## Supporting information




**Supporting File**: adhm71810‐sup‐0001‐SuppMat.docx.

## Data Availability

The data that support the findings of this study are available from the corresponding author upon reasonable request.
