# Supplementary material for: Protein‐Polymer Dynamic Hydrogels Utilizing Native Ligand Binding for Injectable Therapeutic Delivery and 3D Printing
Source: Adv Healthc Mater. 2026 Oct 9;15(41):e71810. doi: 10.1002/adhm.71810 (PMC13740944; doi:10.1002/adhm.71810)
Supplement: Supplementary file 1 [file ADHM-15-0-s001.docx]

***Supporting Information***

**Protein-Polymer Dynamic Hydrogels Utilizing Native Ligand Binding for Injectable Therapeutic Delivery and 3D Printing**

*S. Cem Millik^1^, Yonghui Wang^2^, Kefan Song^2^, Jessica M. Snyder^3^, LeAnn Le^4^, Lauren G. Brown^1^, Shanny Hsuan Kuo^3^, Ashleigh B. Theberge^1,5^, Suzie H. Pun^2,4^, and Alshakim Nelson^1,4^**

^1^ Department of Chemistry, University of Washington, Seattle, WA, USA

^2^ Department of Bioengineering, University of Washington, Seattle, WA, USA

^3^ Department of Comparative Medicine, University of Washington, Seattle, WA, USA

^4^ Molecular Engineering and Sciences Institute, University of Washington, Seattle, WA, USA

^5^ Department of Urology, University of Washington, Seattle, WA, USA

* Correspondence: Alshakim Nelson ([alshakim@uw.edu](mailto:alshakim@uw.edu))

**Table of Contents**

1. Materials	3

2. Instrumentation	3

3. Synthesis of Hyperbranched Poly(glycerol) (HPG)	4

3.1. Purification and Handling of Reagents	4

3.2. Synthesis and Purification of HPG	4

Figure S1	5

4. Synthesis and Purification of Palmitate-Modified HPG (HPG-C16)	5

5. Synthesis and Purification of Soluble Albumin Nanoparticles (SANs)	6

6. Dynamic Light Scattering (DLS)	7

7. Circular Dichroism (CD) Spectroscopy of SANs and BSA	8

8. Preparation of HPG- and SAN-Based Hydrogels	8

8.1. Preparation of Pre-Saturated SAN Hydrogels	9

Figure S2	9

9. Rheometry of Hydrogels	9

Figure S3	10

Figure S4	11

Figure S5	12

Figure S6	13

Figure S7	14

Figure S8	16

Figure S9	17

10. Hydrogel Injection Force Measurements	18

Figure S10	19

11. Direct Ink Writing (DIW)	19

12. Hydrogel Dissolution Experiments	19

13. In Vitro IgG Release Experiments	20

13.1. IgG Dye Labeling	20

13.2. Monitoring In Vitro IgG Release	20

14. In Vivo Release and Biocompatibility Experiments	21

14.1. Hydrogel Preparation for In Vivo Experiments	21

14.2. Injections and IVIS Imaging	21

Figure S11	22

Figure S12	23

14.3. Evaluation of Biocompatibility	23

Table S1	24

Table S2	24

Table S3	25

15. Statistical Analysis	26

**1. Materials**

Bovine serum albumin (BSA; BSAS 1.0) was purchased from Bovogen Biologicals. Immunoglobulin G from bovine serum (IgG; I5506), glycidol (G5809), glutaraldehyde solution (G7526; 8.7% w/w), calcium hydride (CaH_2_; 21170), 1,4-dioxane (296309; anhydrous), *N*,*N*-dimethylformamide (DMF; 227056; anhydrous), *N*-methyl-2-pyrrolidone (NMP; 328634; anhydrous), 1,1,1-tris(hydroxymethyl)propane (TMP; 148083; 99.8%), potassium methoxide solution (CH_3_OK; 60402; 25.1% in MeOH), palmitoyl chloride (P78; 98.6%), *N*,*N*-diisopropylethylamine (DIPEA; D125806; 99.8%), sodium phosphate monobasic monohydrate (71507), sodium phosphate dibasic dihydrate (71643), ethanolamine (398136), hydrochloric acid (HX0603), sodium borohydride (NaBH_4_; 213462), sodium azide (NaN_3_; S2002), Dowex 50WX8 hydrogen-form resin (44509), acetone (179124), diethyl ether (Et_2_O; 673811), methanol-*d*_4_ (CD_3_OD; 441384), Antifoam A concentrate (A5633), 3-[(3-cholamidopropyl)dimethylammonio]-1-propanesulfonate (CHAPS; 220201), and Pluronic® F-127 (F-127; P2443) were purchased from MilliporeSigma. Molecular sieves (MK449004; grade 564) were purchased from VWR International. Methanol (MeOH; A452-1), sodium chloride (S271), propylene glycol (ICN15195790), phosphate-buffered saline 10X solution (PBS; BP399-1), DMEM (31053028), and FluoroBrite DMEM (A1896701) were purchased from Fisher Scientific. Sulfo-Cyanine5 NHS ester (NHS-sulfo-Cy5; 23320) was purchased from Lumiprobe.

**2. Instrumentation**

Proton nuclear magnetic resonance (^1^H NMR) spectra were obtained using a Bruker AVANCE series instrument operating at a 500 MHz frequency. Dynamic light scattering (DLS) data were obtained with a Zetasizer Nano ZS using a quartz cuvette (Starna Scientific). Rheometry was performed using a TA Instruments Discovery Hybrid Rheometer-2 with an Advanced Peltier Plate system. Circular dichroism (CD) spectra were obtained with a Jasco J-1500 CD spectrophotometer, using a quartz cuvette (FireflySci) with a 0.5 mm path length. Spectrophotometry (UV-vis) was performed using a Thermo Scientific NanoDrop One spectrophotometer and BioTek Cytation 5 cell imaging multimode reader. Fluorometry was performed using a BioTek Cytation 5. Injection force testing was performed using a TestResources 100 Series Universal Test Machine with 1.1 kN and 44 N load cells, compression fixtures, and a custom syringe holder (Figure S10). In vivo fluorescence imaging was performed using a Xenogen IVIS Spectrum.

**3. Synthesis of Hyperbranched Poly(glycerol) (HPG)**

**3.1. Purification and Handling of Reagents**

Prior to the HPG synthesis, glycidol was dried over CaH_2_ for 18 h, vacuum-distilled, and degassed by the freeze-pump-thaw method. The purified glycidol was stored at 5 °C until use. Anhydrous dioxane and NMP, once opened (i.e., stored with perforated seals), were dried over molecular sieves for 24 h and sparged with N_2_ before use. TMP was dried under vacuum prior to weighing. All other reagents were used as received and stored according to manufacturer recommendations, unless otherwise specified.

**3.2. Synthesis and Purification of HPG**

A 250 mL 2-neck round-bottom Schlenk flask was flame-dried under vacuum. TMP (0.450 g; 3.35 mmol) was added to the flask. MeOH (400 µL) was then added over the TMP in the flask, followed by CH_3_OK solution (260 µL; 0.885 mmol; 25.1% in MeOH). All additions were performed under positive pressure of N_2_. After the TMP had dissolved, the flask was briefly swirled to homogenize the mixture. Bulk MeOH in the mixture was evaporated with N_2_ flow through a bleed needle. After the TMP mixture appeared solid, residual MeOH was removed under vacuum (~ 3 Pa) over 18 h at 21 °C. Note: the TMP was not heated under vacuum to avoid sublimation. Following removal of the MeOH, an oven-dried overhead stirrer assembly was promptly attached to the flask under positive pressure of N_2_. Anhydrous dioxane (45 mL) was then charged to the flask. The flask was preheated using an aluminum flask carrier at a nominal temperature of 115 °C. PTFE tubing (20 gauge) was then connected to the flask through a needle (14 gauge) and septum under positive pressure of N_2_. The needle was removed from the septum, and using a syringe pump, glycidol (45 mL; 674 mmol) was added through the PTFE tubing over 24 h, at 115 °C, under N_2_, while stirring at ~ 200 rpm. Following completion of glycidol addition, the reaction was allowed to proceed for an additional 24 h under the same conditions. Monomer consumption was confirmed by ^1^H NMR spectroscopy. The reaction mixture was then cooled, bulk dioxane was aspirated, and MeOH (100 mL) was added to dissolve the product. Dowex 50WX8 hydrogen-form resin (~ 1 cc) was finally added to protonate the product. After 15 min, the Dowex resin was removed by centrifugation, and the product was precipitated in acetone. Precipitation in acetone was repeated once. The product was then dissolved in deionized H_2_O at 10–15 wt% and dialyzed (25 kDa MWCO) against deionized H_2_O for 3 d at 21 °C. Following dialysis, the purified HPG in aqueous solution was filtered with a 0.45 µm polyethersulfone filter and stored at ~ 25 wt% at 5 °C until use. Purity was confirmed by ^1^H NMR spectroscopy in CD_3_OD, and appropriate size was confirmed by DLS. Percent yield was ~ 80%. ^1^H NMR (500 MHz, CD_3_OD, 25 °C): δ = 3.94–3.53 (br; 1H, 2H; HPG).

**Figure S1.** Inverse-gated ^13^C NMR spectrum (500 MHz, CD3OD, 25 °C) of HPG.

**4. Synthesis and Purification of Palmitate-Modified HPG (HPG-C16)**

An aqueous solution of HPG (~ 60 mL; ~ 25 wt%) was transferred to a 250 mL round-bottom Schlenk flask. The HPG solution was frozen using liquid N_2_ and lyophilized in the flask over 48 h. The flask containing the HPG was then heated under vacuum (95 °C; ~ 3 Pa) for 18 h to remove trace water. The mass of this dry HPG was determined to be 14.8 g (222 mmol hydroxyl). Data from prior experiments using size-exclusion chromatography with multi-angle light scattering and inverse-gated ^13^C NMR spectroscopy were used to determine a hydroxyl content for high-molecular-weight HPGs of 15 mmol g^−1^; this value was used to calculate equivalents of hydroxyl groups of the HPG. Anhydrous NMP (100 mL) was added over the dried HPG, and the HPG was dissolved at 50 °C. Following dissolution of the HPG, the reaction mixture was allowed to cool to 21 °C, and DIPEA (510 µL; 2.93 mmol) was added. Next, PTFE tubing (20 gauge) was connected to the flask through a needle (14 gauge) and septum under positive pressure of N_2_. The needle was removed from the septum, and using a syringe pump, palmitoyl chloride solution (20.6 mL; 2.27 mmol; 3.33% v/v in anhydrous NMP) was added through the PTFE tubing over 1 h, at 21 °C, under N_2_, while stirring at 400 rpm. Following complete addition of the palmitoyl chloride, the reaction mixture was heated to 40 °C and was allowed to proceed for ~ 20 h. The reaction mixture was then transferred to dialysis tubing (25 kDa MWCO) and was dialyzed against MeOH for ~ 24 h at 21 °C. During dialysis, some material initially precipitated from solution but largely redissolved after four changes of the dialysate. Following dialysis, a small amount of remaining insoluble material was separated via centrifugation for 10 min at 3000 *g* and discarded. Following centrifugation, the solution of crude product was concentrated using a rotary evaporator and precipitated in acetone. Three additional precipitations were performed: once more in acetone and twice in Et_2_O. After each precipitation, the product was redissolved in minimal MeOH. Following the final precipitation, the product was isolated as solid grains and dried under vacuum (~ 3 Pa) over several days. Purity and functionalization were confirmed by ^1^H NMR spectroscopy in CD_3_OD. Functionalization was determined relative to the HPG repeat unit using ^1^H NMR spectroscopy. A factor of 1.1, corresponding to the average number of hydroxyl groups per repeat unit, previously determined from experiments with inverse-gated ^13^C NMR spectroscopy and size-exclusion chromatography with multi-angle light scattering, was used to estimate the degree of functionalization relative to hydroxyl groups, *f*_n_ = 0.70%. Appropriate size was confirmed by DLS. Percent yield was ~ 95%. ^1^H NMR (500 MHz, CD_3_OD, 25 °C): δ = 3.94–3.53 (br; 1H, 2H; HPG), 2.37 (br, 2H), 1.63 (br, 2H), 1.30 (br, 2H), 0.91 (t, 3H).

**5. Synthesis and Purification of Soluble Albumin Nanoparticles (SANs)**

Lyophilized BSA powder (2.00 g) was dissolved in 0.35 M phosphate buffer (58.69 mL; pH ~ 7.4 at 37 °C; *I* = 1.00 M, adjusted with NaCl) to afford a 0.5 mM solution of BSA. This solution was filtered with a 0.22 µm syringe filter (cellulose acetate). The filtered solution (50 mL; 0.025 mmol) was then added to a 250 mL round-bottom flask equipped with a PTFE stir bar. The flask was lowered into a water bath at 37 °C, Antifoam A solution (20 µL; 25% w/w in propylene glycol) was added to the BSA solution, and the solution was stirred for 20 min at 250 rpm. Using a syringe pump, glutaraldehyde solution (8.7% w/w in H_2_O) was added over 3 h at 37 °C, while stirring at 250 rpm. Depending on the target size of the SAN, different addition rates were used to add different amounts of glutaraldehyde over 3 h. For SAN-30, an addition rate of 0.377 mL h^−1^ was used (1.13 mL solution; 1.00 mmol glutaraldehyde). For SAN-45, an addition rate of 0.425 mL h^−1^ was used (1.27 mL solution; 1.13 mmol glutaraldehyde). For SAN-60, an addition rate of 0.471 mL h^−1^ was used (1.41 mL solution; 1.25 mmol glutaraldehyde). Note: the source, age, and storage conditions for glutaraldehyde were found to be important for synthesizing SANs reproducibly in the nominal sizes. Some calibration of reaction conditions, especially between lots of glutaraldehyde, could be necessary. Additionally, careful adjustment of the syringe pump and verification of accurate dispensing is essential, since relatively small differences in the quantity of glutaraldehyde added can result in significant differences in sizes of the SANs. Immediately following complete addition of glutaraldehyde, aqueous ethanolamine hydrochloride (2.5 M) was added (4.00 mL, 10 mmol for SAN-30; 4.50 mL, 11.3 mmol for SAN-45; 5.00 mL, 12.5 mmol for SAN-60). The reaction was allowed to proceed under the same conditions (37 °C, 250 rpm) for 1 h. Next, aqueous NaBH_4_ (1.0 M) was added (2.5 mL, 2.5 mmol for SAN-30; 2.8 mL, 2.8 mmol for SAN-45; 3.1 mL, 3.1 mmol for SAN-60). After 30 min, an equivalent portion of NaBH_4_ was added, and the reaction was allowed to proceed under the same conditions (37 °C, 250 rpm) for another 30 min. Note: NaBH_4_ was pre-weighed and was rapidly dissolved in deionized H_2_O immediately before addition to the reaction mixture. A bleed needle was used to vent the reaction vessel following the addition of NaBH_4_. The reaction mixture was incubated at 5 °C for ~ 20 h, stirring at 80 rpm. For purification, the reaction mixture was dialyzed (25 kDa MWCO) against deionized H_2_O for 3 d at 5 °C. Following dialysis, the SAN solution was filtered using a 1 µm syringe filter (glass fiber), frozen using liquid N_2_, and lyophilized over 3 d. SANs were stored at −20 °C or −80 °C until use. Appropriate size was confirmed by DLS. Typical percent yield was ~ 90%.

**6. Dynamic Light Scattering (DLS)**

For DLS of HPG, deionized H_2_O or MeOH was used as the dispersant. For DLS of HPG-C16, MeOH was used as the dispersant. For DLS of BSA and SANs, 1X PBS was used as the dispersant. Samples were filtered with 0.22 µm cellulose acetate syringe filters. DLS experiments were run at 25 °C, using a quartz cuvette. Experiments were conducted using a 173° non-invasive backscatter configuration. Three measurements, consisting of 10 (10 s) runs each, were performed per sample and averaged. Normalized intensity was obtained by dividing each value in the dataset by the maximum value in the dataset.

**7. Circular Dichroism (CD) Spectroscopy of SANs and BSA**

SANs and BSA were dissolved in 25 mM phosphate buffer (pH ~ 7.4) at a concentration of 0.25 mg mL^−1^. Samples were filtered with a 0.22 µm syringe filter (cellulose acetate). CD experiments were run at 25 °C, using a quartz cuvette with a 0.5 mm path length. Spectra were obtained using a digital integration time of 4 s, a scanning speed of 20 nm min^−1^, an accumulation of 4 scans, and a bandwidth of 1 nm. The sample chamber was purged with N_2_ gas for 15 minutes after loading each sample, prior to running each experiment. A background spectrum for 25 mM phosphate buffer was recorded and subtracted from the spectrum for each material. Normalized spectra were obtained by dividing each value in the dataset by the square root of the sum of the squares of each value in the dataset.

**8. Preparation of HPG- and SAN-Based Hydrogels**

Hydrogels were generally prepared by adding solid components to one syringe and liquid components (1X PBS unless specified otherwise) to another, connecting both syringes with a 90° elbow connector, and mixing the components by pushing the contents of the syringes back and forth between the two syringes. Contents of the syringes were dissolved and homogenized over 3 d. The connected syringes were stored at 5 °C during this time, and their contents were mixed briefly every few hours over the course of the 3 d. Finally, when the contents of the syringes appeared homogeneous, the contents were transferred to a single syringe, the syringe was capped, and it was centrifuged for 30 s at 1000 *g* with the tip of the syringe oriented upward to aid in the removal of excess air from the syringes. Note: care was taken to use minimal centrifugal force to avoid leakage from syringes. Capped syringes with excess air removed were additionally centrifuged tip-down in 10 min intervals at 3000 *g* to remove small bubbles when necessary. Hydrogels were stored in capped syringes at 5 °C until use.

For example, to prepare 2.0 g of a 15 wt% hydrogel with a 3:2 mass ratio of SAN-45 to HPG-C16, HPG-C16 (0.12 g) was first added to a syringe with the plunger removed, followed by SAN-45 (0.18 g), before the plunger was reattached. The plunger was removed from a second syringe, an elbow connector was attached, and the elbow connector was capped. 1X PBS (1.7 mL) was carefully added to this capped syringe without spillage into the elbow connector. The plunger was reattached, and the syringe was inverted and quickly shaken in a single motion to remove PBS from the tip of the syringe. The cap could then be removed from the elbow connector without risk of expelling PBS from the slightly pressurized system. The two syringes were then connected, their contents were dissolved and homogenized, and excess air and small bubbles were removed, as described above.

**8.1. Preparation of Pre-Saturated SAN Hydrogels**

Pre-saturation of SANs with palmitate was accomplished by preparing stock solutions of the SANs, mixing them with an excess of powdered palmitate (50 mg palmitate for ~ 2.8 mL SAN solution), incubating the mixtures with orbital shaking (21 °C, 24 h, 150 rpm), and filtration with a 0.22 µm syringe filter (cellulose acetate). Pre-saturated SAN stock solutions (as opposed to solids) were used to prepare the pre-saturated SAN hydrogels. Standard (unsaturated) SAN hydrogels were prepared analogously but without free palmitate for direct comparison.

**Figure S2.** 15-(3:2)-45 hydrogel, pre-saturated with free palmitate (sat.) and unsaturated (unsat.).

**9. Rheometry of Hydrogels**

Hydrogels for rheometry were prepared as described above. Particular care was taken to remove bubbles prior to testing. For all experiments, a cross-hatched 20 mm stainless steel geometry was used, with a cross-hatched lower plate, and a solvent trap to minimize evaporation. The shear (test) gap was set to 750 µm, with a trim offset of 25 µm. Samples were conditioned at 25 °C, using a 5 min pre-shear interval at a shear rate of 0.1 s^−1^, followed by equilibration for 5 min. Logarithmic shear rate sweeps in the range of 0.1–100 s^−1^ were performed at 25 °C to determine apparent viscosities of the hydrogels, as well as to probe apparent shear-thinning behavior. Power-law index *n* was determined by fitting a power-law model to the manually selected power-law region of each flow curve. Oscillatory strain amplitude sweeps in the range of 0.1– 100% were performed at a constant angular frequency of 10 rad s^−1^, at 25 °C, to determine the limit of the linear viscoelastic regime. The limit of the linear viscoelastic regime in terms of stress amplitude was taken as the yield stress. Oscillatory frequency sweeps in the range of 0.1–100 rad s^−1^ were performed at a constant strain amplitude within the linear regime (1%), at 25 °C, to probe time-dependent viscoelastic behavior of the hydrogels. Multi-step oscillatory tests, with alternating small-amplitude and large-amplitude steps, were used to investigate thixotropy and self-healing behavior of the hydrogels. For these tests, seven steps (60 s each), with alternating high (200%) and low (1%) strain amplitude, at a constant angular frequency of 10 rad s^−1^, at 25 °C, were used. Tests with longer (600 s) steps were also performed. Finally, temperature ramps from 5–50 °C, at a rate of 2 °C min^−1^, were performed at a constant strain amplitude of 1% and a constant angular frequency of 10 rad s^−1^. For all experiments, at least three runs were performed per sample and averaged.

**Figure S3.** Rheology of 30 wt% F-127 hydrogel. **(a)** Steady-shear viscosity and stress curves (mean; *n* = 3). *η* ≈ 660 Pa s at *γ^·^* = 1.0 s^−1^; power-law index *n* ≈ 0.010. **(b)** Frequency sweep data (mean; *n* = 6). *G'* ≈ 19400 Pa at *ω* = 10 rad s^−1^; tan *δ* ≈ 0.053 at *ω* = 10 rad s^−1^. **(c)** Stress amplitude sweep data (mean; *n* = 3). *σ*_y_ ≈ 610 Pa at *ω* = 10 rad s^−1^. **(d)** Multi-step high- and low-amplitude oscillatory tests showing self-healing and thixotropy (mean; *n* = 3).

**Figure S4.** Additional rheology of 15 wt% HPG-C16 hydrogel. **(a)** Stress amplitude sweep data (mean; *n* = 3). *σ*_y_ ≈ 130 Pa at *ω* = 10 rad s^−1^. **(b)** Temperature ramp data (mean; *n* = 3). **(c)** Steady-shear viscosity and stress curves (mean; *n* = 3). *η* ≈ 140 Pa s at *γ^·^* = 1.0 s^−1^; power-law index *n* ≈ −0.32.

**Figure S5.** Rheology of mixtures with a 1:1 mass ratio of HPG-C16 to BSA—unsaturated (unsat.) and pre-saturated with free palmitate (sat.). Bar charts show *η* at *γ^·^* = 1.0 s^−1^, *G'* and tan *δ* at *ω* = 10 rad s^−1^, and *σ*_y_ at *ω* = 10 rad s^−1^. **(a)** Vial inversion test of 15 wt% mixtures with unsat. BSA (left) and sat. BSA (right). **(b)** Steady-shear viscosity and stress curves for 15 wt% mixtures (mean; *n* = 6). **(c)** Steady-shear viscosity and stress curves for 30 wt% mixtures (mean; *n* = 6 for unsat., *n* = 3 for sat.). **(d)** Frequency sweep data for 15 wt% mixtures (mean; *n* = 3). **(e)** Frequency sweep data for 30 wt% mixtures (mean; *n* = 3). **(f)** Stress amplitude sweep data for sat. mixtures (mean; *n* = 3; unsat. mixtures did not show yielding behavior at *ω* = 10 rad s^−1^). **(g)** Comparison of *η* and power-law index *n* for each mixture (mean ± SD; *n* = 6 for all mixtures except 30 wt% sat., for which *n* = 3). **(h)** Comparison of *G'* and tan *δ* for each mixture (mean ± SD; *n* = 3). **(i)** Comparison of *σ*_y_ for sat. mixtures (mean ± SD; *n* = 3).

**Figure S6.** Steady-shear viscosity curves for 15 wt% mixtures of BSA or SAN with HPG or HPG-C16 (mean; *n* = 3 for all mixtures except BSA + HPG-C16, for which *n* = 6).

**Figure S7.** Systematic rheological evaluation of HPG-C16 + SAN hydrogels with respect to total concentration, ratio of components, and SAN size. **(a)** Tested hydrogels in syringes. The hydrogel in the center in each set of three gels is the 15-(3:2)-45 formulation. **(b)** Steady-shear viscosity and stress curves with total concentration varied (mean; *n* = 3). **(c)** Steady-shear viscosity and stress curves with ratio of components varied (mean; *n* = 3). **(d)** Steady-shear viscosity and stress curves with SAN size varied (mean; *n* = 3). **(e)** Frequency sweep data with total concentration varied (mean; *n* = 3). **(f)** Frequency sweep data with ratio of components varied (mean; *n* = 3). **(g)** Frequency sweep data with SAN size varied (mean; *n* = 3). **(h)** Stress amplitude sweep data with total concentration varied (mean; *n* = 3). **(i)** Stress amplitude sweep data with ratio of components varied (mean; *n* = 3). **(j)** Stress amplitude sweep data with SAN size varied (mean; *n* = 3).

**Figure S8.** Additional rheology of 15-(3:2)-45 and 15-(1:1)-60 hydrogels. Bar charts show *η* at *γ^·^* = 1.0 s^−1^, *G'* and tan *δ* at *ω* = 10 rad s^−1^, and *σ*_y_ at *ω* = 10 rad s^−1^. **(a)** Stress amplitude sweep data (mean; *n* = 3). **(b)** Temperature ramp data (mean; *n* = 3). **(c)** Steady-shear viscosity and stress curves (mean; *n* = 3). **(d)** Comparison of *η* and power-law index *n* (mean ± SD; *n* = 3). **(e)** Comparison of *G'* and tan *δ* (mean ± SD; *n* = 3). **(f)** Comparison of *σ*_y_ (mean ± SD; *n* = 3). **(g)** Comparison of *G'* and tan *δ* at 21 °C and 37 °C (mean ± SD; *n* = 3). **(h)** Long-interval multi-step high- and low-amplitude oscillatory tests showing self-healing and thixotropy.

**Figure S9.** Summary data from rheological evaluation of HPG-C16 + SAN hydrogels containing SANs that were left unsaturated (unsat.) or were pre-saturated with free palmitate (sat.). Bar charts show *η* at *γ^·^* = 1.0 s^−1^, *G'* and tan *δ* at *ω* = 10 rad s^−1^ and *ω* = 0.1 rad s^−1^, and *σ*_y_ at *ω* = 10 rad s^−1^. **(a)** Comparison of *η* and power-law index *n* for unsat. and sat. 15-(3:2)-45 gels (mean ± SD; *n* = 3). **(b)** Comparison of *G'* and tan *δ* at moderate (*ω* = 10 rad s^−1^) and low (*ω* = 0.1 rad s^−1^) *ω* for unsat. and sat. 15-(3:2)-45 gels (mean ± SD; *n* = 3). **(c)** Comparison of *σ*_y_ for unsat. and sat. 15-(3:2)-45 gels (mean ± SD; *n* = 3). **(d)** Comparison of *η* and power-law index *n* for unsat. and sat. 15-(1:1)-60 gels (mean ± SD; *n* = 3). **(e)** Comparison of *G'* and tan *δ* at moderate and low *ω* for unsat. and sat. 15-(1:1)-60 gels (mean ± SD; *n* = 3). **(f)** Comparison of *σ*_y_ for unsat. and sat. 15-(1:1)-60 gels (mean ± SD; *n* = 3). **(g)** Conceptual depiction of a hypothetical pre-saturated HPG-C16 + SAN dynamic nanocolloidal network.

**10. Hydrogel Injection Force Measurements**

A custom syringe holder (Figure S10) was designed and 3D printed to interface with the compression fixtures of a TestResources 100 Series Universal Test Machine and hold a 1 mL syringe (BD, 309628) with the tip of the syringe oriented downward. Tests were performed with 23 G and 27 G needles of 0.5 in length. Hydrogels were prepared as described above. A slight excess of hydrogel was loaded into the syringe prior to each test. A preload of 0.1 N was used for each test. An injection rate of 1.2 mL min^−1^ (68 mm min^−1^) was used over a test period of 10 s. For all experiments, three runs were performed and averaged. Plateau injection force *F*_p_ was determined by fitting an exponential plateau model to the injection force data (GraphPad Prism 10). Second-order smoothing with 10 neighbors was applied to the injection force data for visualization (GraphPad Prism 10).

**Figure S10.** Injection force measurement setup comprising a 3D-printed syringe holder attached to a load frame fitted with compression fixtures.

**11. Direct Ink Writing (DIW)**

Direct ink writing was performed on a HyRel Engine SR extrusion-based 3D printer equipped with an SDS-5 syringe dispenser. Standard 5 mL syringes (BD) were used with 27 G needles with 0.5 in length. An extrusion rate of 70 pulses µL^−1^, a print speed of 200 mm min^−1^, and a layer height of 0.21 mm were used for all prints.

**12. Hydrogel Dissolution Experiments**

Materials for dissolution experiments were prepared as described above. Each material was prepared using the corresponding dissolution medium. Dissolution media were 1X PBS and DMEM with 10% FBS and 1% penicillin-streptomycin. NaN_3_ (50 mM) was included in the dissolution media to prevent microbial contamination. Each material (300 mg) was loaded into a 1.5 mL microcentrifuge tube (VWR, 89000-028) and centrifuged for 1 min at 3000 *g* to uniformly localize the material to the bottom of the tube. Dissolution medium (1.0 mL) was placed over each material. Tubes were incubated at 37 °C without agitation. Time points for sampling were 0.5 d, 1.5 d, 3.5 d, 7.5 d, 15.5 d, and 31.5 d. At each time point, the supernatant was carefully collected from each tube and replaced with 1.0 mL of fresh dissolution medium. Collected supernatants were frozen with liquid N_2_ and lyophilized. Mass loss from materials due to dissolution was calculated from dry mass remaining after lyophilization, assuming concentrations of the components of the dissolution media remained constant. Three replicates were included for each material.

**13. In Vitro IgG Release Experiments**

**13.1. IgG Dye Labeling**

Lyophilized bovine IgG powder was reconstituted in 1X PBS to a concentration of ~ 5 mg mL^−1^. Aliquots (1.0 mL) of the IgG stock solution were stored at −20 °C until use. NHS-sulfo-Cy5 (5 mg) was dissolved in anhydrous DMF to prepare a stock solution (10 mM) and stored air-free at −20 °C until use. IgG solution (4.7 mL; 0.14 µmol) was transferred to a glass vial. The IgG solution was diluted twofold with 0.1 M phosphate buffer (pH ~ 7.4) with 20% v/v DMSO and 4% w/v CHAPS. NHS-sulfo-Cy5 solution (80 µL; 0.80 µmol) was added to the IgG solution and promptly mixed in. The reaction was allowed to proceed for 6 h at 21 °C, with orbital shaking at 150 rpm. The reaction mixture was refrigerated for 18 h. The crude IgG-Cy5 product was purified by diafiltration (Amicon Ultra-15; 50 kDa MWCO) against 75 mL of 1X PBS with 10% v/v DMSO followed by 75 mL of 1X PBS. The pure IgG-Cy5 was concentrated to ~ 5 mg mL^−1^ in 1X PBS and stored at 5 °C until use within 3 d. Cy5-containing mixtures were shielded from light whenever possible. Degree of labeling with the Cy5 dye was determined by spectrophotometry (i.e., using absorbance at 280 nm and 646 nm) to be ~ 2.0 M M^−1^. Percent yield was ~ 90%.

**13.2. Monitoring In Vitro IgG Release**

Materials containing IgG-Cy5 (1 mg mL^−1^) were prepared for in vitro release experiments, as described above, using 1X PBS with NaN_3_ (50 mM) to prevent microbial contamination. During material preparation, the IgG-Cy5 was added in a stock solution with 1X PBS. The release medium was FluoroBrite DMEM with 10% FBS, 1% penicillin-streptomycin, and 50 mM NaN_3_. Each material (300 mg) was loaded into a 1.5 mL microcentrifuge tube (VWR, 89000-028) and centrifuged for 1 min at 3000 *g* to uniformly localize the material to the bottom of the tube. Release medium (1.0 mL) was placed over each material. Tubes were incubated at 37 °C without agitation. Time points for sampling were 0.5 d, 1.5 d, 3.5 d, 7.5 d, 15.5 d, and 31.5 d. At each time point, the supernatant was carefully collected from each tube and replaced with 1.0 mL of fresh release medium. Quantities of IgG-Cy5 were determined by measuring fluorescence intensity of the supernatants and using a standard curve obtained using time-matched (in terms of incubation at 37 °C) standard solutions. Three replicates were included for each material.

**14. In Vivo Release and Biocompatibility Experiments**

**14.1. Hydrogel Preparation for In Vivo Experiments**

For animal studies, hydrogels containing IgG-Cy5 (1 mg mL^−1^) were prepared as described above, using sterile 1X PBS (without NaN_3_). Hydrogels and their components were prepared aseptically; prior to mixing, all components were filtered in solution with a 0.22 µm syringe filter (cellulose acetate) and isolated and stored aseptically.

**14.2. Injections and IVIS Imaging**

All animal studies were approved by, and performed in accordance with, the University of Washington Institutional Animal Care and Use Committee (protocol number: 4053-01). Female SKH1-Elite mice (strain code: 477) aged 6–8 weeks were purchased from Charles River Laboratories. Groups were randomly assigned. Investigators were not blinded to the experimental groups. No animals were excluded from the study. Prior to the start of experiments, animals were allowed to acclimate to the laboratory environment for 5 d. Four animals per group were subcutaneously injected, in the right dorsal caudal flanks, with 100 µL of a hydrogel or 1X PBS containing 100 µg of IgG-Cy5. Injections were performed under brief isoflurane anesthesia. Imaging was performed under brief isoflurane anesthesia, and mice were weighed, once per day on days 0–4, 6, 7, 9, 11, 14, 16, 18, and 21. In vivo fluorescence (IVIS) images were obtained using an exposure time of 1 s, a binning setting of medium, an F/Stop setting of 4, an excitation filter of 640 nm, an emission filter of 680 nm, and a lamp level of low. Black-and-white images were obtained using the auto-exposure setting, a binning setting of medium, and an F/Stop setting of 8. Images were acquired in field of view D. Epifluorescence was quantified in terms of radiant efficiency. For visualization, the color scale was set with a minimum of 6 × 10^8^ p s^−1^ cm^−2^ sr^−1^ µW^−1^ cm^2^ and a maximum of 2 × 10^9^ p s^−1^ cm^−2^ sr^−1^ µW^−1^ cm^2^. At each time point, one animal that received no injection was imaged for comparison. Total radiant efficiency from the whole mouse was quantified using a rectangular 4 cm × 7 cm region of interest encompassing the whole mouse except the tail. Total radiant energy efficiency from the injection site was quantified using an elliptical 2.3 cm × 3.3 cm region of interest centered on the injection site.

**Figure S11.** In vivo fluorescence (IVIS) images showing IgG release over 21 d, from subcutaneously injected gels in the right dorsal caudal flanks of mice. The first animal pictured in the 1X PBS group received no injection.

**Figure S12.** Quantification of total radiant efficiency from the whole mouse over 21 d (mean ± SD; *n* = 4).

**14.3. Evaluation of Biocompatibility**

On day 22, mice were euthanized via CO_2_ asphyxiation. Immediately following euthanasia, cardiocentesis was performed for complete blood count and serum chemistry on three mice per treatment group. Mice for blood collection were selected either randomly or on the basis of whether a sufficient quantity of blood could be obtained. Blood was only collected from two mice in Group 1 (control group), due to the difficulty in obtaining a sufficient quantity of blood. Following blood collection, gross necropsies were performed on four mice per group, and tissues were collected for histopathological evaluation by a board-certified veterinary pathologist. Tissues were fixed in 10% neutral-buffered formalin. Histology was performed on three mice per group (selected randomly). Tissues examined as part of the histopathological evaluation included skin and subcutaneous tissue harvested from the injection site, lung, heart, liver, kidney, spleen, and mesenteric fat with pancreas. Tissues were routinely paraffin-embedded, sectioned at 4 µm, and slides were stained with hematoxylin and eosin. Images of representative lesions were captured from glass slides using NIS-Elements BR 64-bit and plated in Adobe Photoshop 2025. Image white balance, brightness, and contrast were adjusted using auto corrections applied to the entire image. Original magnification is stated, and a scale bar is placed.

**Table S1.** Body condition score (BCS), body mass (BM), and gross necropsy results (*n* = 4).

| **Group** | **Median BCS** | **Mean BM [g]** | **Injection site** | **Spleen** | **GI** | **Liver** | **Kidney** | **Heart** | **Lungs^1^** |
| --- | --- | --- | --- | --- | --- | --- | --- | --- | --- |
| Group 1  [1X PBS] | 2.5 | 19.9 | NSF | NSF | NSF | NSF | NSF | NSF | Mottled red (4/4) |
| Group 2  [15 wt% HPG-C16] | 3 | 24.4 | NSF | 1.5× enlarged (2/4) | NSF | NSF | NSF | NSF | Mottled red (3/4); hemorrhagic (1/4) |
| Group 3  [15-(3:2)-45] | 3 | 22.6 | NSF | 1.5× enlarged (3/4) | NSF | NSF | NSF | NSF | Mottled red (2/4); diffusely pink (2/4) |
| Group 4  [15-(1:1)-60] | 3 | 25.7 | NSF | 1.5–2× enlarged (4/4) | NSF | NSF | NSF | NSF | Mottled red (2/4); hemorrhagic (1/4) |

NSF = no significant findings; GI = gastrointestinal; ^1^The mottled red color to the lungs and hemorrhage was presumed in all groups to be secondary to euthanasia and cardiocentesis.

**Table S2.** Histologic findings in skin and subcutaneous tissues at the injection site (*n* = 3).

| **Group*** | **Incidence and severity of macrophage infiltrates** | **Extension into deep dermis** |
| --- | --- | --- |
| Group 1  [1X PBS] | 0/3 | – |
| Group 2  [15 wt% HPG-C16] | 3/3 moderate to marked | 2/3 |
| Group 3  [15-(3:2)-45] | 1/3 moderate to marked; 2/3 mild to moderate | 2/3 |
| Group 4  [15-(1:1)-60] | 1/3 moderate to marked; 1/3 moderate; 1/3 mild | 2/3 |

*Neutrophils and multinucleated giant cells were present in the dermis in all groups to a similar degree, often centered on hair fragments and follicles, and most likely related to a background strain effect in the SKH1-Elite hairless mice.

**Table S3.** Serum chemistry and complete blood count data from individual mice, obtained via postmortem cardiocentesis.

| **Animal*** | **1-1** | **1-4** | **2-2** | **2-3** | **2-4** | **3-1** | **3-2** | **3-3** | **4-1** | **4-2** | **4-4** |
| --- | --- | --- | --- | --- | --- | --- | --- | --- | --- | --- | --- |
| **ALP [U L^−1^]** | 82 | 88 | 54 | 170 | 96 | 103 | 73 | 94 | 101 | 98 | 87 |
| **AST [U L^−1^]** | 131 | 92 | 205 | 240 | 74 | 2048 | 174 | 137 | 72 | 610 | 112 |
| **ALT [U L^−1^]** | 49 | 48 | 44 | 101 | 40 | 1842 | 118 | 80 | 39 | 503 | 43 |
| **CK [U L^−1^]** | 204 | 111 | 636 | 505 | 84 | 396 | 93 | 111 | 71 | 622 | 206 |
| **Albumin [g dL^−1^]** | 3.9 | 4 | 3.7 | 4.1 | 3.7 | 3.5 | 3.9 | 3.5 | 3.5 | 3.6 | 3.6 |
| **TBIL [mg dL^−1^]** | 0.4 | 0.3 | 0.4 | 0.3 | 0.2 | 0.3 | 0.3 | 0.2 | 0.2 | 0.3 | 0.3 |
| **TP [g dL^−1^]** | 6 | 6.1 | 5.8 | 6.8 | 6 | 5.6 | 6.2 | 5.5 | 5.7 | 5.8 | 5.7 |
| **Globulin [g dL^−1^]** | 2.1 | 2.1 | 2.1 | 2.7 | 2.3 | 2.1 | 2.3 | 2 | 2.2 | 2.2 | 2.1 |
| **BUN [mg dL^−1^]** | 21 | 23 | 26 | 19 | 22 | 20 | 23 | 18 | 15 | 20 | 17 |
| **TC [mg dL^−1^]** | 91 | 82 | 91 | 119 | 80 | 74 | 97 | 82 | 82 | 85 | 77 |
| **Glucose [mg dL^−1^]** | 202 | 132 | 140 | 141 | 233 | 268 | 157 | 258 | 189 | 271 | 155 |
| **Calcium [mg dL^−1^]** | 10.3 | 10.6 | 9.8 | 11.3 | 10.9 | 10 | 10.2 | 10.4 | 11 | 11.2 | 10 |
| **PHOS [mg dL^−1^]** | 15.1 | 16.3 | 15.2 | 20.6 | 18.9 | 17.8 | 17.4 | 14.7 | 16.7 | 18.6 | 15.9 |
| **TCO_2_ [mmol L^−1^]** | 13 | 16 | 14 | 17 | 19 | 19 | 18 | 19 | 22 | 20 | 20 |
| **A/G Ratio** | 1.9 | 1.9 | 1.8 | 1.5 | 1.6 | 1.7 | 1.7 | 1.8 | 1.6 | 1.6 | 1.7 |
| **Hemolysis** | 1+ | 1+ | 1+ | 1+ | N | N | N | N | 1+ | N | N |
| **Lipemia** | N | N | N | N | N | N | N | N | N | N | N |
|  |  |  |  |  |  |  |  |  |  |  |  |
| **WBC [× 10^−3^ µL^−1^]** | 5.9 | 6.4 | 4.2 | 4.4 | 8.1 | 7.7 | 4.9 | 10** | 5 | 8.4 | 6 |
| **RBC [× 10^−6^ µL^−1^]** | 11.5 | 10.99 | 9.42 | 12.59 | 10.31 | 9.58 | 10.42 | 10.12 | 10.47 | 10.68 | 10.04 |
| **HGB [g dL^−1^]** | 17.8 | 17.4 | 17.3 | 19.5 | 16.5 | 15.5 | 17 | 16.4 | 17 | 17 | 16.1 |
| **HCT [%]** | 53 | 58 | 52.2 | 57 | 59.6 | 55.7 | 58.1 | 58.4 | 59.8 | 56 | 57.4 |
| **MCV [fL]** | 46 | 53 | 55 | 45 | 58 | 58 | 58 | 58 | 57 | 52 | 57 |
| **MCH [pg]** | 15.5 | 15.8 | 18.4 | 15.5 | 16 | 16.2 | 16.3 | 16.2 | 16.2 | 15.9 | 16 |
| **MCHC [g dL^−1^]** | 33.6 | 30 | 33.1 | 34.2 | 27.7 | 27.8 | 29.3 | 28.1 | 28.4 | 30.4 | 28 |
| **PLT [× 10^−3^ µL^−1^]** | 531 | 734 | 435 | 1157 | 787 | 755 | 282 | 760 | 426 | 730 | 393 |
| **Neutrophil [µL^−1^]** | 826 | 1139 | 798 | 1082 | 1215 | 924 | 1274 | 2240** | 795 | 1571 | 966 |
| **Band [µL^−1^]** | 0 | 0 | 0 | 0 | 0 | 0 | 0 | 0 | 0 | 0 | 0 |
| **Lymphocyte [µL^−1^]** | 4720 | 4832 | 3360 | 3036 | 6642 | 6083 | 3430 | 7040** | 3965 | 6073 | 4164 |
| **Monocyte [µL^−1^]** | 354 | 358 | 42 | 242 | 243 | 616 | 147 | 540 | 180 | 613 | 528 |
| **Eosinophil [µL^−1^]** | 0 | 38 | 0 | 31 | 0 | 77 | 49 | 170 | 60 | 134 | 330 |
| **Basophil [µL^−1^]** | 0 | 32 | 0 | 9 | 8 | 0 | 0 | 10 | 0 | 8 | 12 |

N = normal; ALP = alkaline phosphatase; AST = aspartate aminotransferase; ALT = alanine aminotransferase; CK = creatine kinase; TBIL = total bilirubin; TP = total protein; BUN = blood urea nitrogen; TC = total cholesterol; PHOS = phosphorus; TCO_2_ = bicarbonate (total CO_2_); A/G = albumin/globulin; WBC = white blood cell; RBC = red blood cell; HGB = hemoglobin; HCT = hematocrit; MCV = mean corpuscular volume; MCH = mean corpuscular hemoglobin; MCHC = mean corpuscular hemoglobin concentration; PLT = platelet; *Each animal is designated by [Group #]-[Animal #]. **Animal 3-3 had chronic active inflammation at the heart base consistent with arteritis.

**15. Statistical Analysis**

Data were reported as mean ± standard deviation (SD) unless specified otherwise. Normalized intensity for DLS was obtained by dividing each value in the dataset by the maximum value in the dataset. Normalized CD spectra were obtained by dividing each value in the dataset by the square root of the sum of the squares of each value in the dataset. Power-law index *n* was determined by fitting a power-law model to the manually selected power-law region of each flow curve. Ordinary one-way ANOVA in conjunction with Tukey’s multiple comparisons test with a single pooled variance was used to assess statistical significance in the summary rheometric data (GraphPad Prism 10). Statistical significance was defined as *p* < 0.05. Sample size (*n*) was three for each analysis. *F*_p_ was determined by fitting an exponential plateau model to the injection force data (GraphPad Prism 10). Second-order smoothing with 10 neighbors was applied to the injection force data for visualization (GraphPad Prism 10).
